# The PAR complex controls the spatiotemporal dynamics of F-actin and the MTOC in directionally migrating leukocytes

**DOI:** 10.1242/jcs.146217

**Published:** 2014-10-15

**Authors:** Carolina Lage Crespo, Claudio Vernieri, Philipp J. Keller, Massimiliano Garrè, Jeffrey R. Bender, Joachim Wittbrodt, Ruggero Pardi

**Affiliations:** 1 Division of Immunology, Transplantation and Infectious Diseases, San Raffaele Scientific Institute, 20132 Milan, Italy; 2 IFOM Foundation, Institute FIRC of Molecular Oncology, 20139 Milan, Italy; 3 Janelia Farm Research Campus, Howard Hughes Medical Institute, Ashburn, 20147 VI, USA; 4 Department of Medicine, Raymond and Beverly Sackler Foundation Cardiovascular Laboratory, Yale University, New Haven, 06511 CT, USA; 5 Center for Organismal Studies Heidelberg, University of Heidelberg, 69120 Heidelberg, Germany; 6 Vita-Salute San Raffaele University School of Medicine, 20132 Milan, Italy

**Keywords:** 3D Migration, Leukocyte, Inflammation, Cell polarity, Cytoskeleton, PAR complex

## Abstract

Inflammatory cells acquire a polarized phenotype to migrate towards sites of infection or injury. A conserved polarity complex comprising PAR-3, PAR-6 and atypical protein kinase C (aPKC) relays extracellular polarizing cues to control cytoskeletal and signaling networks affecting morphological and functional polarization. However, there is no evidence that myeloid cells use PAR signaling to migrate vectorially in three-dimensional (3D) environments *in vivo*. Using genetically encoded bioprobes and high-resolution live imaging, we reveal the existence of F-actin oscillations in the trailing edge and constant repositioning of the microtubule organizing center (MTOC) to direct leukocyte migration in wounded medaka fish larvae (*Oryzias latipes*). Genetic manipulation in live myeloid cells demonstrates that the catalytic activity of aPKC and the regulated interaction with PAR-3 and PAR-6 are required for consistent F-actin oscillations, MTOC perinuclear mobility, aPKC repositioning and wound-directed migration upstream of Rho kinase (also known as ROCK or ROK) activation. We propose that the PAR complex coordinately controls cytoskeletal changes affecting both the generation of traction force and the directionality of leukocyte migration to sites of injury.

## INTRODUCTION

Polarization allows cells to sense and to elicit the proper spatiotemporal responses to cues that arise from the surrounding microenvironment. Migrating cells are characterized by a sustained front–rear polarity. At the molecular level, polarized migration involves the establishment and maintenance of a spatial and functional asymmetry of molecular components between the anterior (leading) and posterior (trailing) edges of the migrating cell (Lauffenburger and Horwitz, 1996). How such coordinate partitioning of the cell migration and signaling machinery is controlled and maintained over time is largely unknown. Prevailing models evoke the creation of asymmetry in the distribution of key signaling molecules in the migrating cell, either through the production of rapidly diffusing inhibitory molecules by the front of the cell or through the sequestration of limiting polarity components to the front (Wang, 2009). Hydrostatic-pressure-driven blebbing (Blaser et al., 2006) or local tension (Houk et al., 2012) generated in the leading-edge membrane have been proposed as additional processes underlying polarization and directional migration in selected cell types. A further level of complexity in our understanding of the dynamics of cell polarization is due to the limitations of most *in vitro* models of cell migration, which fail to recreate the heterogeneous three-dimensional (3D) environment in which cells polarize and migrate *in vivo*. Studies performed in 2D substrates can be highly detailed but often provide results that differ substantially from those obtained *in vivo* under 3D conditions (Lämmermann et al., 2008; Pouthas et al., 2008).

In various cell types and species, the PAR complex, consisting of a core of PAR-3, PAR-6 and atypical protein kinase C (aPKC-λ/ι and aPKC-ζ), controls different aspects of cell polarity, ranging from cytoskeletal dynamics to sensing of positional cues. In a close interplay with Rho family members, the PAR complex presides over the architecture of the actin and microtubule cytoskeletons for polarized migration (Iden and Collard, 2008). The small GTPase Cdc42 activates aPKC and the PAR complex through the adaptor molecule PAR-6 (McCaffrey and Macara, 2009). PAR-3, in association with Cdc42–PAR-6–aPKC, binds directly to the Rac1 guanine nucleotide exchange factors (GEFs) Tiam1 and Tiam2, thus mediating Cdc42-induced Rac1 activation and lamellipodia formation (Nishimura et al., 2005).

Whether the PAR polarity complex is functionally required for chemokine-induced leukocyte polarization is unknown. Recent studies have shown that various polarity proteins are differentially localized throughout polarized T cells, suggesting that they might regulate T cell polarization (Gérard et al., 2007; Ludford-Menting et al., 2005). Furthermore, PAR-3 knockdown impairs monocyte migration towards inflammatory signals *in vitro* (Tamehiro et al., 2009).

Using high-resolution live imaging in genetically engineered medaka fish larvae (*Oryzias latipes*), we show that core components of the PAR complex regulate wound-induced directional migration of myeloid cells, through modulation of ROCK-dependent F-actin and microtubule organizing center (MTOC) dynamics. We also found that the catalytic activity of PKC-ζ is required for its own polarized distribution during migration, and that such relocation is a ROCK-independent phenomenon. Thus, we propose that the PAR complex, through the Rho pathway, coordinates the dynamics of both F-actin and the MTOC and microtubules to support the efficient migratory profile of ameboid leukocytes in confined *in vivo* environments.

## RESULTS

### PAR proteins promote the directed migration of myeloid cells *in vivo*

**Fig. 1.**
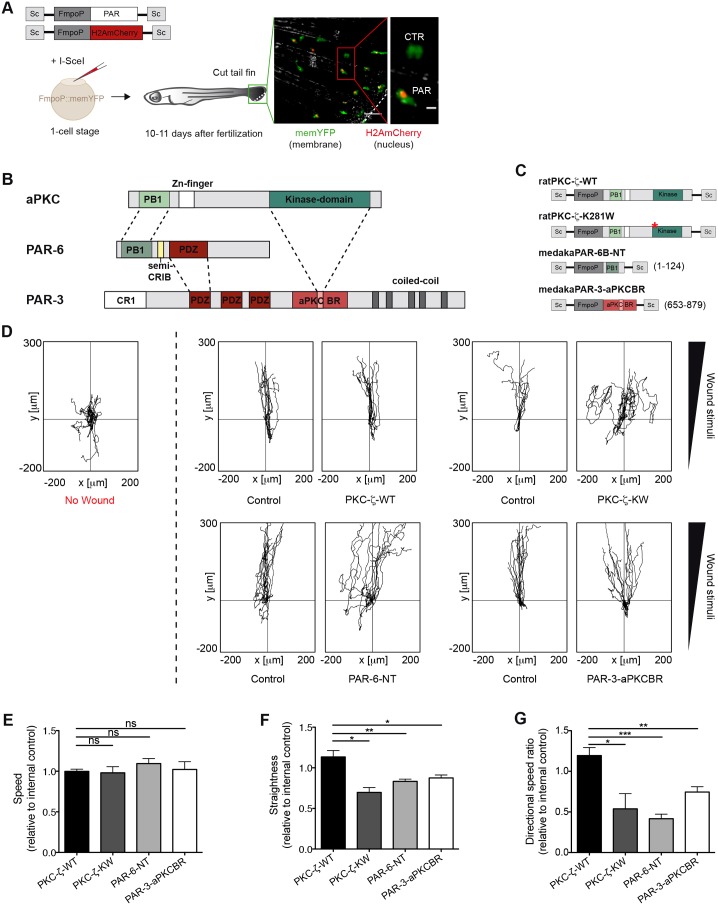
**The PAR complex promotes wound-directed migration of myeloid cells *in vivo*.** (A) TG(FmpoP::memYFP) embryos were injected at the one-cell stage with a 1∶1 mixture of DNA coding for H2AmCherry as a nuclear reporter and one of the PAR transgenes, driven by the myeloid-specific *Fmpo* promoter and flanked by I-*Sce*I integration sites (Sc), in the presence of I-*Sce*I meganuclease. Wounded larvae with mosaic expression of H2AmCherry in tailfin myeloid cells were imaged. The dashed line represents the wound. The inset shows a transgenic cell (Cherry^+^; PAR) and the endogenous control (Cherry^−^; CTR). Scale bars: 50 µm (left panel); 10 µm (inset). (B) Domains of interaction between members of the mammalian PAR complex. Connecting lines indicate regions of the proteins that interact with one another. PB1, phagocyte oxidase/Bem1 domain; Zn, Zinc finger motif; Kinase, catalytic domain; CRIB, Cdc42/Rac interactive binding motif; PDZ, PSD-95/Dlg/Zona occludens-1 domain; CR1, conserved region 1; aPKCBR, aPKC-binding region. A predicted coiled-coil region is also shown. (C) Schematics of the constructs used to perturb the function of the PAR complex in myeloid cells. Numbers refer to amino acid positions. *K to W mutation at codon 281. NT, N-terminal domain. (D) 2D tracks of individual leukocytes migrating in the tailfin of unwounded fish (left panel) or towards the tailfin wound (right panels). No wound, *n* = 11; control/PKC-ζ-WT, *n* = 12; control/PKC-ζ-KW, *n* = 10; control/PAR-6-NT, *n* = 13; control/PAR-3-aPKCBR, *n* = 13). Tracks are from one representative experiment of at least three independent experiments. (E–G) Quantification of 2D (E) speed, (F) path straightness and (G) directional speed ratio of myeloid cells during the wound response. Data are expressed as the mean±s.e.m. of at least three separate experiments (PKC-ζ-WT, *n* = 27 cells in three larvae; PKC-ζ-KW, *n* = 27 cells in three larvae; PAR-6-NT, *n* = 45 cells in four larvae; PAR-3-aPKCBR, *n* = 64 cells in five larvae); *P<0.05; **P<0.01; ***P<0.001; ns, non-significant (two-tailed unpaired Student's *t*-test). See also supplementary material Fig. S1; Movies 1, 2.

To determine how PAR-3, PAR-6 and aPKC regulate the directed migration of leukocytes *in vivo* in a 3D environment, we developed a model of wound-induced inflammatory cell migration in medaka fish, based on live imaging of tissue-resident myeloid cells expressing membrane-tethered YFP [memYFP, using the transgenic line TG(FmpoP::memYFP) as, in medaka, myeloperoxidase (MPO) is expressed in mixed myeloid lineages that also contain sudanophilic material; supplementary material Fig. S1A] (Aghaallaei et al., 2010; Grabher et al., 2007). Because silencing of the PAR components affects the morphogenesis of several embryonic tissues in zebrafish (Horne-Badovinac et al., 2001; Munson et al., 2008; Wei et al., 2004) and the use of morpholinos is not effective in juvenile medaka (∼9–11 days post-fertilization), we adopted a meganuclease-driven, transient transgenesis approach based on the injection of embryos at the one-cell stage (Rembold et al., 2006). We devised a strategy whereby a set of PAR-complex-interfering mutants, expressed under the myeloid-cell-specific *Fmpo* promoter (FmpoP), were co-injected with a nuclear-localized fluorescent marker (mCherry fused to histone H2A – H2AmCherry), to track the PAR-mutant-expressing cells (Fig. 1A–C) (Souren et al., 2009). Using this established gene expression system, we first determined that the two transgenes were coexpressed in >72% of cells (supplementary material Fig. S1B–D). As the transgenes were expressed in a mosaic fashion, we could directly compare the control subpopulation (H2AmCherry^−^) with the PAR-mutant-expressing subset (H2AmCherry^+^), to assess migration-associated parameters during the wound-response within the same animal (Fig. 1A).

To interfere with the PAR-complex-associated catalytic activity, we ectopically expressed the kinase-inactive PKC-ζ-K281W (PKC-ζ-KW) mutant or wild-type PKC-ζ (PKC-ζ-WT) as a control, specifically in myeloid cells (Fig. 1C). Ectopic expression of wild-type PKC-ζ in myeloid cells did not alter wound-directed migration in terms of the ‘straightness’ of the path and directional speed (Fig. 1D,F–G). By contrast, PKC-ζ-KW-expressing cells, even if fully motile, moved more randomly, in a ‘zig-zag’ fashion, towards the wound, indicating that the catalytic activity of aPKC is dispensable for mobilisation of the cells, but promotes persistent migration *in vivo* (Fig. 1D–G; supplementary material Movies 1, 2). These findings were confirmed using multicistronic viral 2A peptide-based vector, whereby PKC-ζ variants and the fluorescent reporter mCherry were stoichiometrically coexpressed in all cells as independent proteins (supplementary material Fig. S1E–H).

Next, we evaluated whether aPKC was promoting leukocyte migration as part of its molecular and functional interaction with PAR-3 and PAR-6. To this end, we ectopically expressed either the aPKC-binding region of medaka PAR-3 (PAR-3-aPKCBR) or the N-terminal domain of medaka PAR-6B (PAR-6-NT) specifically in myeloid cells (Fig. 1B,C), as overexpression of these deletion mutants has been shown to compete for the binding of endogenous aPKC with PAR-3 or PAR-6, respectively (Nakayama et al., 2008; Nishimura et al., 2005). Consistent with the PKC-ζ-KW data, both PAR-3-aPKCBR- and PAR-6-NT-expressing cells still sensed the migration-inducing cues but moved less directly to the wound site, thus suggesting that PAR-3 and PAR-6 contribute, together with aPKC, to the migrating response of leukocytes to the wound (Fig. 1D–G). Taken together, these findings establish that the functional integrity of the PAR-6–aPKC–PAR-3 complex promotes the wound-directed migration of leukocytes *in vivo*.

### Defined spatial and temporal patterns of F-actin and MTOC dynamics occur during leukocyte directional migration *in vivo*

**Fig. 2.**
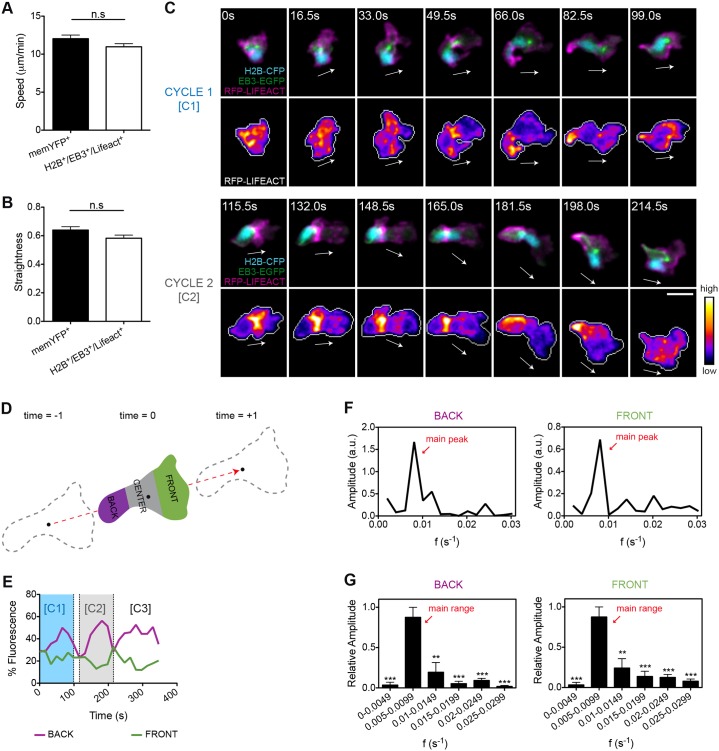
**F-actin shows an oscillatory anteroposterior polarity during wound-directed migration of leukocytes *in vivo*.** (A,B) Quantification of 2D (A) speed and (B) path straightness of memYFP^+^ or H2B–CFP^+^ EB3–EGFP^+^ RFP–Lifeact^+^ leukocytes migrating to wounds. Data are expressed as the mean±s.e.m. of all analyzed cells (memYFP^+^, *n* = 75 cells in three larvae; H2B–CFP^+^ EB3–EGFP^+^ RFP–Lifeact^+^, *n* = 64 cells in three larvae); ns, non-significant; two-tailed unpaired Student's *t*-test. (C) Two consecutive cycles (C1 and C2) of front-to-back F-actin waves (lower panels) were visualized in transgenic larvae coexpressing RFP–Lifeact and EB3–EGFP in myeloid cells [TG(FmpoP:: EB3–EGFP/FmpoP::RFP–Lifeact)], following transient expression of H2B–CFP as a nuclear marker (upper panels). Shown are frames from representative movies of myeloid cells migrating in the wounded tailfin. The white arrows indicate the direction of migration. Cell outlines are indicated by the gray line. Scale bar: 10 µm. (D) Schematics of the 2D geometric compartmentalization used to quantify F-actin subcellular distribution. Black dots indicate the cell centroid shown at three consecutive time-points, and the dashed red arrow indicates the direction of migration vector. (E) Timecourse of F-actin front and back fluorescence signal distribution in the cell depicted in C. Cycle C1 and C2 are those visualized in C. (F) Fourier spectra showing the time frequencies that compose the oscillatory signal of F-actin in the back and front regions of the cell shown in C. The peak with the highest amplitude value (main peak) corresponds to the predominant frequency of oscillation. Frequencies with lower amplitude values are also displayed and give a minor contribution to the oscillatory signal. a.u., arbitrary units. (G) Histograms showing the relative contribution of ranges of frequencies to back and front F-actin oscillations. Note that a predominant range of oscillatory frequencies (main range) emerges from ranges of secondary frequencies. Data are expressed as the mean±s.e.m. of all analyzed cells (*n* = 8 cells in four larvae); statistical analysis was performed between the main range and each of the remaining ranges of frequencies; **P<0.01; ***P<0.001 (two-tailed paired Student's *t*-test). See also supplementary material Fig. S2; Movie 4.

PAR proteins control cell polarity by establishing and maintaining asymmetry in the localization of both the actomyosin contractile system and microtubules (Iden and Collard, 2008). To perform simultaneous imaging and quantification of both F-actin and microtubule dynamics in the living animal, we generated a double transgenic line – TG(FmpoP::EB3-EGFP/FmpoP::RFP-Lifeact) – in which medaka myeloid cells expressed simultaneously Ruby-tagged Lifeact (RFP–Lifeact) (Riedl et al., 2008), which detects polymerized actin, and the end-binding protein 3 (EB3) fused to EGFP, which binds to microtubule plus-end tips and also allows visualization of the MTOC. By applying the meganuclease system in this transgenic line, we expressed histone H2B fused to CFP (H2B–CFP), to allow visualization of nuclei in directionally migrating cells. Cells that were triply positive for RFP–Lifeact, EB3–EGFP and H2B–CFP migrated towards the wound as efficiently as memYFP-positive cells (Fig. 2A,B) and displayed a highly variable and dynamic subcellular localization of the visualized cytoskeletal structures (supplementary material Movie 3).

To search for recurrent patterns in F-actin dynamics linked to migration, we examined and quantitatively assessed the fluorescence intensity of RFP–Lifeact along the entire length of leukocytes during the migration process (Fig. 2D) (Solecki et al., 2009). Interestingly, F-actin displayed clear oscillatory patterns in migrating cells, with a temporally defined alternating enrichment at the front and back edges (Fig. 2C,E; supplementary material Movie 4). Ratiometric analysis of RFP–Lifeact versus membrane-tethered YFP signals confirmed that these oscillations were not an artifact due to convolution of the plasma membrane, which can be particularly problematic in 3D environments (supplementary material Fig. S2). To better characterize F-actin oscillatory behavior and to circumvent the high variability in fluorescence levels between individual cells, we performed Fourier analysis (Geva-Zatorsky et al., 2010). Fourier analysis decomposes oscillatory signals into their basic frequencies and associated amplitudes. The outcome is the Fourier spectrum, which shows the contribution, by means of amplitude, of each frequency to the oscillatory signal. Different oscillatory signals can be therefore compared based on their frequency components and associated relative contributions. Surprisingly, we found that both front and back F-actin waves displayed a main oscillation frequency common to all analyzed cells and that reflected their consistency. This oscillation frequency was centered between 0.005 and 0.0099 s^−1^ (oscillation period between 1.85 and 3.33 min) (Fig. 2F,G; supplementary material Movie 4). Notably, periodic accumulation of F-actin in the cell uropod paralleled boosts of speed in the migrating leukocyte. By contrast, F-actin enrichment in the leading edge was linked to low-speed movement associated with lamellipodia extension and spatial exploration of the 3D context by the migrating cells (supplementary material Fig. S2). The robustness of the Fourier spectra in different cells suggests that periodic localization of F-actin in the cellular front and back during migration does not occur by chance, but is a conserved mechanism that can have a functional role in proper migration.

**Fig. 3.**
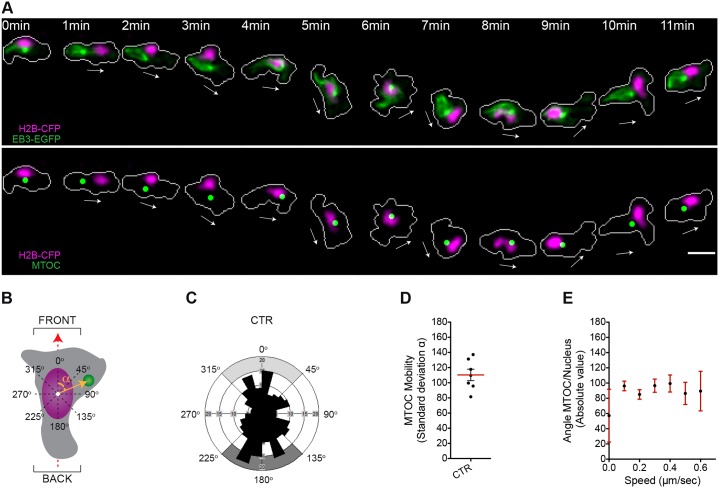
**The MTOC is highly mobile in the perinuclear compartment of leukocytes migrating to wounds *in vivo*.** (A) The microtubules and the nucleus are visualized in a migrating leukocyte generated as described for Fig. 2. Upper panel, fluorescence images; lower panel, green dots represent a digitalized reconstruction of the MTOC position. The white arrows indicate the direction of migration. Cell outlines are shown as gray lines. Scale bar: 10 µm. (B) Schematic of the 2D analysis of MTOC perinuclear positioning during migration. The dashed red arrow indicates the direction of migration defined as in Fig. 2. The yellow arrow originating from the nuclear centroid (white dot) to the MTOC is the MTOC–nucleus vector. The angle *α* between the MTOC–nucleus vector and the direction-of-migration vector is the MTOC-nucleus angle (orange arc). (C) Rose diagram mapping the MTOC orientation and the respective spatial frequency of events in migrating myeloid cells (230 counts, seven leukocytes in five larvae). Light or dark gray areas correspond to 90° ranges for front or back orientations, respectively. CTR, control. (D) Quantification of MTOC perinuclear mobility in migrating myeloid cells. MTOC mobility for each cell is represented by the standard deviation associated with the MTOC-nucleus angle during the response to wounding. The red line shows the mean (±s.e.m.) for all cells analyzed (seven leukocytes in five larvae). (E) MTOC perinuclear orientation is plotted against cellular speed. For each range of speed, data represents the mean±s.e.m. (226 counts, seven leukocytes in five larvae); R Spearman = 0.008, *P* (two-tailed) = 0.8932 (non-significant). See also supplementary material Fig. S3A–D; Table S1; Movie 5.

To assess the MTOC positioning during migration in our *in vivo* model, we determined the angular positioning of the MTOC around the nucleus during wound-induced directional migration. We assigned a front or back orientation when the MTOC was positioned within the 315°–45° or 135°–225° angular sections, respectively, as determined based on the vectorial axis of migration (Fig. 3B). The analysis of individual cells migrating towards the wound showed that the MTOC was highly dynamic and continuously shifted from the front to the back of the nucleus in directionally migrating cells (f events*_front_* = 0.27; f events*_back_* = 0.34) (Fig. 3A,C,D; supplementary material Table S1; Movie 5). This mobility pattern might be caused by a marked anterior to posterior nuclear displacement combined with a strong rotational movement of both the MTOC and the nucleus (supplementary material Fig. S3A–D). Finally, and unlike F-actin waves, positioning of the MTOC either at the front or at the back of the nucleus did not correlate with overall speed rates of the migrating leukocytes (Fig. 3E).

### Regulation of anteroposterior polarity of F-actin by PAR proteins

**Fig. 4.**
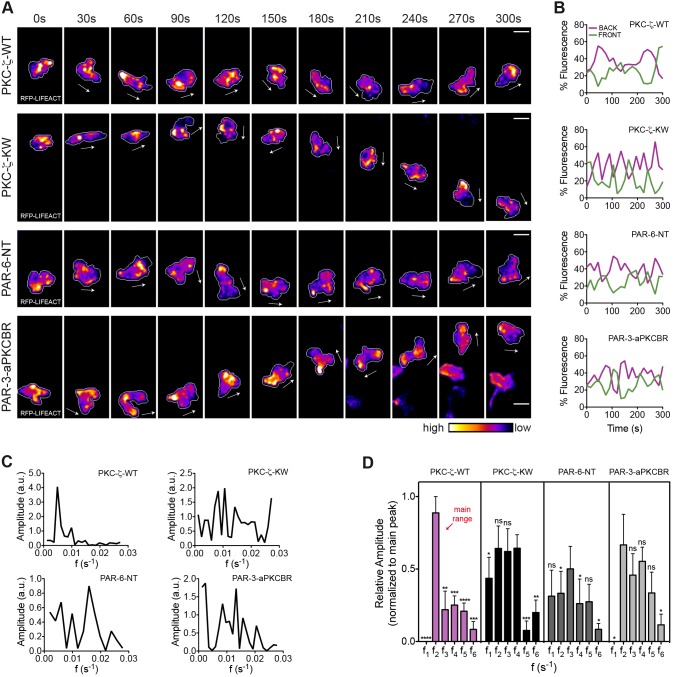
**The PAR complex regulates the anteroposterior polarity of F-actin during wound-directed migration of leukocytes *in vivo*.** (A) F-actin distribution is visualized in transgenic larvae with RFP–Lifeact and EB3–EGFP in myeloid cells, following transient coexpression of nuclear H2B–CFP and each of the indicated PAR transgenes. Frames from representative movies of migrating leukocytes in the wounded tailfin are shown. The white arrows indicate the direction of migration. Outlines of cells are shown as a gray line. Scale bars: 10 µm. (B) Timecourse of F-actin front and back fluorescence intensity distribution in the cells shown in A. (C) Fourier spectra of F-actin oscillations in the back region of the cells depicted in A. a.u., arbitrary units. (D) Comparison of histograms of F-actin oscillatory frequencies in the back region of the cell [ranges (s^−1^) are the same as shown in Fig. 2G]. Data are expressed as the mean±s.e.m. of all analyzed cells (PKC-ζ-WT, eight leukocytes in five larvae; PKC-ζ-KW, eight leukocytes in six larvae; PAR-6-NT, seven leukocytes in four larvae; PAR-3-aPKCBR, six leukocytes in four larvae); *P<0.05; **P<0.01; ***P<0.001; ****P<0.0001; ns, non-significant (two-tailed paired Student's *t*-test). See also supplementary material Fig. S4; Movie 6.

The observation that enrichment of F-actin in the uropod occurs when ameboid leukocytes increase speed rates raises the possibility that actin polymerization in the trailing edge functions as an oscillatory engine that cyclically propels the cell forward. In agreement with such a mechanism, we found a negative relationship between F-actin enrichment and nuclear positioning (supplementary material Fig. S3E,F). Hence, we tested whether in our model expression of dominant-interfering mutants of PAR complex components affects the oscillatory pattern of rear F-actin in the wound-directed migration of myeloid cells. We preliminarily determined that ectopic expression of wild-type PKC-ζ in myeloid cells did not alter the periodicity of F-actin waves in the uropod. Fourier spectra of the F-actin signal in the cell rear displayed a dominant frequency of oscillation centered between 0.005 and 0.0099 s^−1^, which was comparable to that of control cells. By contrast, Fourier profiles of PKC-ζ-KW, PAR-6-NT and PAR-3-aPKCBR cells did not show a clearly dominant frequency of oscillation, indicative of profound modifications in the nature of these oscillatory signals when the PAR complex is functionally perturbed (Fig. 4; supplementary material Movie 6). Moreover, in contrast to the homogeneity of PKC-ζ-WT waves, we observed a huge variability between cells expressing mutant versions of single PAR proteins, suggesting that the robustness of physiological oscillations was disrupted upon interference with the PAR complex. Taken together, our data suggest that the three core components of the PAR complex are coordinating the dynamics of actin polymerization in the rear of leukocytes migrating rapidly *in vivo* and that the catalytic function of PKC-ζ is required for this process.

As an indicator of morphological polarization, we analyzed cell shape changes (by means of cellular roundness) in myeloid cells expressing the various PAR mutants as they migrated towards the wound. First, we observed that the shape profile of wild-type PKC-ζ leukocytes was typically migratory and was comparable to that of control cells [roundness_(0.4–0.6)_: control = 42%, PKC-ζ-WT = 48%; roundness of control = 0.5351±0.0010, roundness of PKC-ζ–WT = 0.5403±0.0089; ±s.e.m.]. Next, we found that PKC-ζ-KW and PAR-6-NT cells adopted more rounded shapes, possibly owing to multiple random protrusions (roundness of PKC-ζ-KW = 0.5940±0.0080; roundness of PAR-6-NT = 0.6243±0.0099; ±s.e.m.). Surprisingly, cells expressing the aPKCBR of medaka PAR-3 showed minimal changes on mean roundness values (roundness of PAR-3-aPKCBR = 0.5494±0.0093; ±s.e.m.) but, instead, revealed a wide range of shape changes, from extremely elongated to more rounded phenotypes (supplementary material Fig. S4; Table S2).

### Regulation of MTOC perinuclear dynamics by PAR proteins

**Fig. 5.**
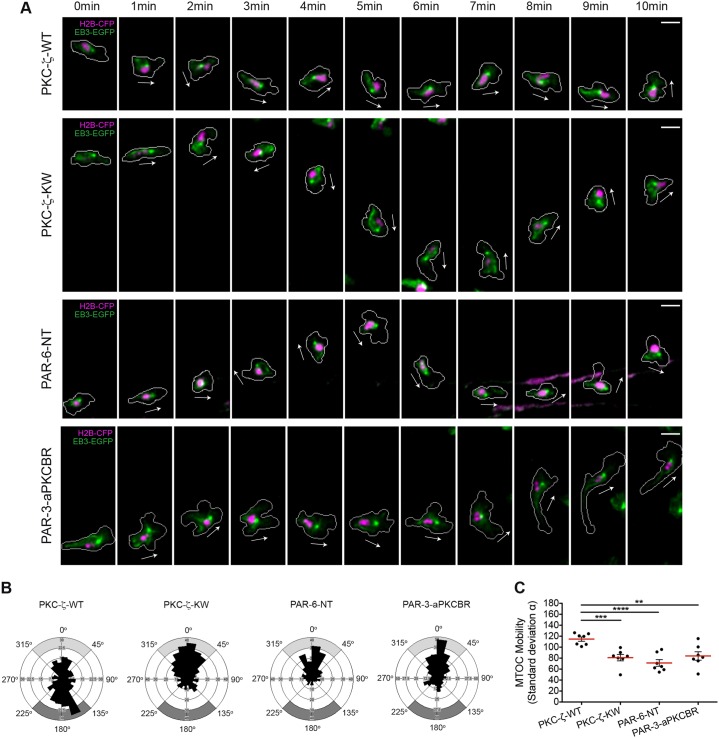
**The PAR complex regulates the MTOC perinuclear positioning during wound-directed migration of leukocytes *in vivo*.** (A) The microtubules and the nucleus are visualized in migrating leukocytes generated as described for Fig. 4. The white arrows indicate the direction of migration. Cell outlines are shown as gray lines. Scale bars: 10 µm. (B) Rose diagrams mapping the orientation of the MTOC and the respective spatial frequency in migrating myeloid cells [PKC-ζ-WT, 265 counts (seven leukocytes in six larvae); PKC-ζ-KW, 347 counts (seven leukocytes in five larvae); PAR-6-NT, 235 counts (seven leukocytes in five larvae); PAR-3-aPKCBR, 307 counts (seven leukocytes in five larvae)]. Gray areas indicate front or back orientation as in Fig. 3. (C) Comparison of MTOC perinuclear mobility in migrating myeloid cells as described in Fig. 3D (PKC-ζ-WT, seven leukocytes in six larvae; PKC-ζ-KW, seven leukocytes in five larvae; PAR-6-NT, seven leukocytes in five larvae; PAR-3-aPKCBR, seven leukocytes in five larvae); **P<0.01; ***P<0.001; ****P<0.0001 (two-tailed unpaired Student's *t*-test). See also supplementary material Table S1; Movie 7.

Prior reports have identified PAR components as key regulators of MTOC mobility and spatial localization in migrating cells (Etienne-Manneville and Hall, 2001; Etienne-Manneville and Hall, 2003; Gomes et al., 2005; Schmoranzer et al., 2009; Solecki et al., 2009; Solecki et al., 2004). Hence, we assessed whether perturbation of the PAR complex interferes with MTOC perinuclear dynamics in our *in vivo* model of wound-directed 3D migration. As with the analysis of F-actin dynamics, ectopic expression of wild-type PKC-ζ in myeloid cells did not alter MTOC mobility around the nucleus. Similar to control cells, the MTOC shifted from front to back with respect to the nucleus during the wound response (PKC-ζ-WT: f events*_front_* = 0.29, f events*_back_* = 0.38). Notably, in cells expressing PKC-ζ-KW, PAR-6-NT and PAR-3-aPKCBR the MTOC was markedly less mobile, and preferentially oriented towards the cell front (PKC-ζ-KW: f events*_front_* = 0.48, f events*_back_* = 0.16; PAR-6-NT: f events*_front_* = 0.53, f events*_back_* = 0.11; PAR-3-aPKCBR: f events*_front_* = 0.48, f events*_back_* = 0.17) (Fig. 5; supplementary material Table S1; Movie 7).

### Rho-dependent actomyosin contractility is required for MTOC mobility around the nucleus in wound-induced myeloid cell migration

**Fig. 6.**
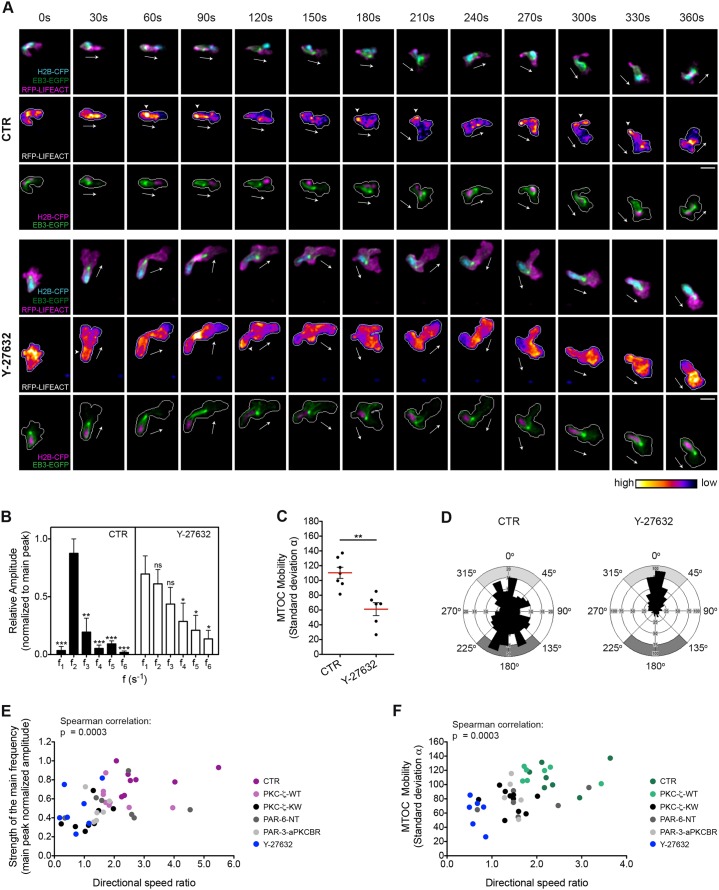
**Rho-kinase-dependent actomyosin contraction is required for MTOC dynamic positioning in leukocytes migrating to wounds *in vivo*.** (A) F-actin (upper and middle panels), the microtubules and the nucleus (upper and lower panels) are visualized in control (CTR) or Y-27632-treated transgenic larvae generated as described for Fig. 2. Frames from representative movies of migrating myeloid cells in wounded tailfins are shown. The white arrows indicate the direction of migration. The arrowheads point to F-actin accumulation at the trailing edge of the cell. Cell outlines are shown as a gray line. Scale bars: 10 µm. (B) Comparison of histograms of F-actin oscillatory frequencies in the back region of the cell [ranges (s^−1^) as shown in Fig. 2G]. Data are expressed as the mean±s.e.m. of all analyzed cells (control, eight leukocytes in four larvae; Y-27632, seven leukocytes in three larvae); *P<0.05; **P<0.01; ***P<0.001; ns, non-significant (two-tailed paired Student's *t*-test). (C) Comparison of MTOC mobility as described for Fig. 3D (control, seven leukocytes in five larvae; Y-27632, six leukocytes in four larvae); **P<0.01 (two-tailed unpaired Student's *t*-test). (D) Rose diagrams mapping the orientation of the MTOC and its spatial frequency [control, 230 counts (seven leukocytes in five larvae); Y-27632, 524 counts (six leukocytes in four larvae)]. Gray areas indicate front or back orientation as in Fig. 3. (E,F) The strength of the main frequency of F-actin oscillations in the back region (E) is plotted against directional speed ratio. Shown is the normalized amplitude of the main frequency peak in the Fourier spectra (44 leukocytes, R Spearman = 0.5149). MTOC perinuclear mobility (F) is plotted against directional speed ratio (41 leukocytes, R Spearman = 0.5416). ***P (two-tailed)<0.001. See also supplementary material Fig. S3E,F; Table S1; Movies 8, 9.

One obvious question that arises from the prior data is whether F-actin, the MTOC and microtubules act coordinately to promote cell migration in leukocytes, as has been previously reported in nocodazole-treated cell lines (Xu et al., 2005) and recently proposed for *Drosophila* hemocytes (Stramer et al., 2010). We did find a link between F-actin distribution and MTOC perinuclear orientation, in that when F-actin was enriched at the uropod the MTOC was behind the nucleus and oriented towards the back of the cell (supplementary material Fig. S3F). To further address how actomyosin contraction functionally contributes to MTOC perinuclear dynamics in migrating leukocytes, we pharmacologically inhibited Rho kinase (also known as ROCK or ROK), the RhoA effector controlling actin–myosin interaction in non-muscle cells, by exposing the larvae to Y-27632. Fourier analysis of F-actin fluctuations in the uropod of the cell confirmed that the periodicity of F-actin movement was profoundly modified upon treatment with Y-27632 (Fig. 6A,B; supplementary material Movie 8). Interestingly, ROCK inhibition with Y-27632 strongly reduced the perinuclear mobility of the MTOC, which became markedly oriented towards the cellular front (Y-27632: f events*_front_* = 0.61, f events*_back_* = 0.11) (Fig. 6A,C,D; supplementary material Table S1; Movie 9). In conclusion, primarily impairing Rho-dependent actomyosin contraction not only alters F-actin localization as expected, but also modifies MTOC mobility around the nucleus. This suggests a direct causal link between these two cytoskeletal networks.

To unveil the functional relevance of the identified F-actin and MTOC dynamics to forward locomotion, we examined the relationships between directional speed and either the periodicity of F-actin oscillations in the cell rear or the mobility of the MTOC in the perinuclear region. Interestingly, we found a statistically significant, positive correlation between the periodicity of F-actin oscillations (as determined by the existence of a dominant peak in Fourier spectra) and the efficiency of directional migration to the wound (Fig. 6E). Likewise, the angular mobility of the MTOC around the nucleus positively correlated with the directional speed of the cell, suggesting that cells need to constantly re-position the MTOC in the perinuclear region to be able to migrate more efficiently towards pro-migratory cues *in vivo* (Fig. 6F).

### The enzymatic activity of PKC-ζ is required to establish and maintain a polarized distribution of the PAR complex

**Fig. 7.**
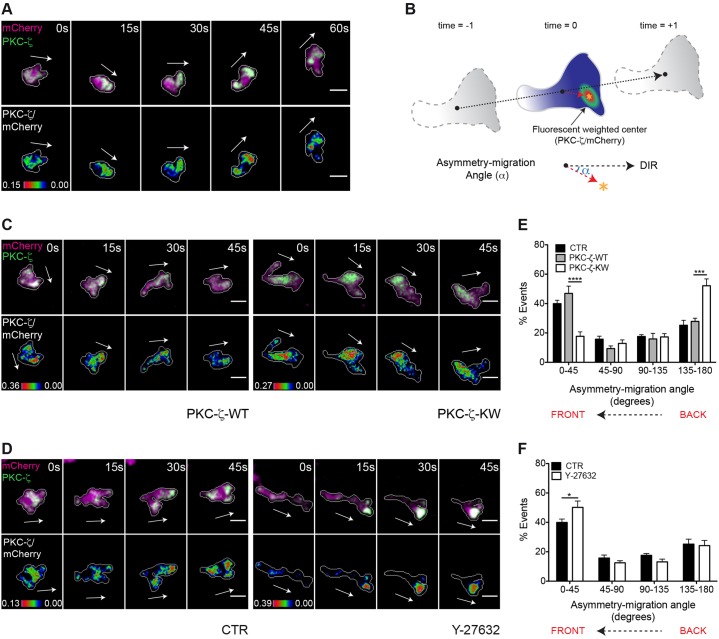
**The catalytic activity of PKC-ζ is essential for its polarized localization in leukocytes migrating *in vivo*.** For A,C and D, Frames from representative movies of migrating myeloid cells in wounded tailfins are shown. The white arrows indicate the direction of migration. Cell outlines are drawn as a gray line. Scale bars: 10 µm. (A) Upper panel, mCherry and GFP–PKC-ζ were visualized in TG(FmpoP::mCherry) larvae transiently expressing GFP–PKC-ζ in myeloid cells. Lower panel, ratiometric GFP–PKC-ζ∶mCherry images were generated using mCherry as a volumetric control. (B) Schematics of the 2D analysis of protein asymmetry during cell migration. Black dots indicate the cell centroid shown at three consecutive time-points, and the dashed black arrow indicates the direction of migration. The asterisk represents the fluorescence center of ratiometric signal, and the dashed red arrow from the cell centroid to the fluorescence center is the asymmetry vector. The angle *α* between the asymmetry vector and the direction of migration (blue arc) is defined as the asymmetry-migration angle, which approaches 180° for a protein that is located at the back of the cell. (C) Upper panel, mCherry and GFP–PKC-ζ were visualized in TG(FmpoP::mCherry) transgenic larvae transiently expressing GFP–PKC-ζ together with PKC-ζ-WT or PKC-ζ-KW in myeloid cells. Lower panel, ratiometric GFP–PKC-ζ∶mCherry images were created. (D) Upper panel, mCherry and GFP–PKC-ζ were visualized in control or Y-27632-treated transgenic larvae established as described for A. Lower panel, ratiometric images GFP–PKC-ζ∶mCherry were generated. (E,F) Histograms show the polarized distribution of ratiometric GFP–PKC-ζ∶mCherry images in migrating cells assessed using the asymmetry-migration angles. CTR, control. Data are expressed as the mean±s.e.m. of all analyzed cells (control, 15 leukocytes in four larvae; PKC-ζ-WT, eight leukocytes in four larvae; PKC-ζ-KW, ten leukocytes in three larvae; Y-27632, 11 leukocytes in four larvae); *P<0.05; ***P<0.001; ****P<0.0001 (two-tailed unpaired Student's *t*-test). See also supplementary material Movies 10, 11.

Neither the spatial and temporal dynamics of PAR complex distribution nor its functional role have been determined in leukocytes migrating *in vivo*. To address this issue, we transiently expressed GFP-tagged PKC-ζ in a transgenic line expressing mCherry as cytoplasmic volume marker [Tg(FmpoP::mCherry)], using the myeloid cell-specific *Fmpo* promoter. Ratiometric imaging of PKC-ζ∶mCherry revealed marked and transient local PKC-ζ increases at the front of migrating cells (Fig. 7A; supplementary material Movie 10). Quantitative analysis of fluorescent protein distribution in ratiometric images (Melichar et al., 2011) confirmed that PKC-ζ localized predominantly at the front of the cell (Fig. 7A,B,D–F).

Next, we investigated the molecular mechanisms underlying PKC-ζ polarization in live leukocytes, based on the hypothesis that the catalytic function of the PAR complex plays a role in such a polarized distribution. To this end, we transiently coexpressed GFP–PKC-ζ with the wild-type or kinase inactive PKC-ζ in TG(FmpoP::mCherry) larvae. Whereas ectopic expression of wild-type PKC-ζ in myeloid cells did not perturb the GFP–PKC-ζ distribution pattern, expression of PKC-ζ-KW led to mislocalization of the fluorescent signal towards the uropod of migrating cells (Fig. 7C,E; supplementary material Movie 11). Thus, activated PKC-ζ is required for its own polarized distribution in leukocytes migrating *in vivo*.

To investigate whether ROCK modulates PAR polarity in leukocytes migrating *in vivo*, we tested the effect of its specific inhibitor Y-27632 on GFP–PKC-ζ subcellular distribution. Notably, GFP–PKC-ζ localized predominantly at the cell front upon treatment with Y-27632, similar to its localization in control cells. Inhibition of ROCK actually increased the degree of front–rear asymmetry of the fluorescence signal (Fig. 7D,F; supplementary material Movie 10). These results indicate that PKC-ζ polarization at the front of the cell is independent of Rho kinase activity when leukocytes migrate to wounds *in vivo*.

### Regulation of RhoA activity by PKC-ζ catalytic function

**Fig. 8.**
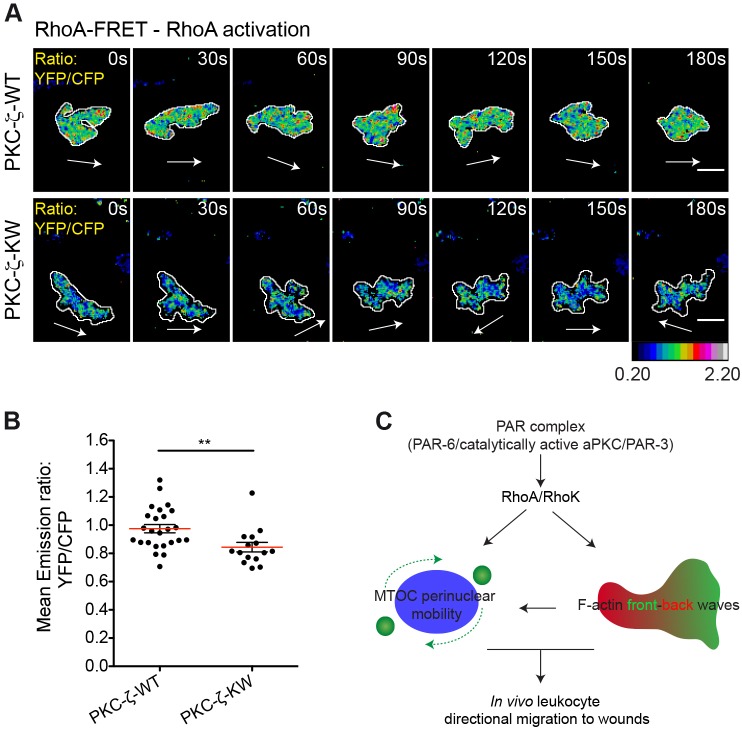
**PKC-ζ regulates RhoA activity in leukocytes migrating to wounds *in vivo*.** (A) Frames from representative movies of migrating myeloid cells in wounded tailfins are shown. The white arrows indicate the direction of migration. Cell outlines are shown as white lines. Scale bars: 10 µm. See also supplementary material Movie 12. RhoA activity was visualized in TG(FmpoP::mCherry) larvae transiently expressing cytosolic RhoA-FRET biosensor together with PKC-ζ-WT or PKC-ζ-KW in myeloid cells. Ratiometric images of YFP∶CFP emission for each cell are shown. (B) Average activation level of RhoA (mean emission ratio of YFP∶CFP for the entire cell during migration) in wound-activated leukocytes. Data are expressed as the mean±s.e.m. of all analyzed cells (PKC-ζ-WT, 25 leukocytes in 15 larvae; PKC-ζ-KW, 15 leukocytes in 5 larvae); **P<0.01 (two-tailed unpaired Student's *t*-test). (C) A model illustrating the mechanism by which the PAR complex controls wound-directed leukocyte migration *in vivo*. The PAR complex coordinately controls Rho-dependent F-actin dynamics and MTOC perinuclear mobility to support the persistent migration of leukocytes to wounds.

Next, we explored whether the catalytic function of the PAR complex modulates RhoA–ROCK signaling in our *in vivo* model of leukocyte migration. To this end, we coexpressed the cytosolic RhoA-FRET (Förster resonance energy transfer) biosensor (Kardash et al., 2010) with the wild-type or the kinase-inactive PKC-ζ variants in medaka leukocytes. FRET ratiometric imaging of the migration process revealed that RhoA activity is reduced in PKC-ζ-KW cells when compared with that of cells expressing wild-type PKC-ζ (Fig. 8A,B; supplementary material Movie 12), indicating that the catalytic activity associated with the PAR complex is upstream of RhoA activation in wound-directed myeloid cell migration.

## DISCUSSION

This study was undertaken to address the *in vivo* relevance of the evolutionarily conserved PAR complex in inflammatory cell polarization and directional motility in response to tissue injury. Our results unveil the relevance of periodic rear-edge enrichment of F-actin in directionally migrating tissue-resident myeloid cells, which is paralleled by a high degree of perinuclear mobility of the MTOC. Both of these features appear to be severely perturbed by the expression of dominant-interfering PAR complex mutants, which results in impaired directionality and cell shape patterns associated with vectorial migration (Fig. 8C). An additional layer of complexity in this model is given by the adaptation of migrating cells to the constraints of the changing 3D environment that they encounter on approaching the wound edge. The geometric complexity of the interstitium induces Cdc42-dependent pathways for leading-edge coordination in chemotactic leukocytes (Lämmermann et al., 2009). One of the most conserved functions of Cdc42 is the activation of aPKC and the PAR module through PAR-6 (McCaffrey and Macara, 2009), thus establishing a potential link between pro-migratory cue sensing and the activation and recruitment of the PAR complex. We found that PKC-ζ activity promotes persistent leukocyte movement, as disruption of its catalytic function increased dispersive motion of cells. The same phenotype was observed in cells expressing mutants interfering with the binding of PAR-6 or PAR-3 to aPKC, although to a variable extent. Despite such differences, the expression of dominant-interfering mutants of PKC-ζ, PAR-6 or PAR-3 affected the same qualitative parameters of leukocyte migration, namely path straightness and directional speed, consistent with the previously shown interdependence of the three core components of the complex in leukocytes migrating directionally to tissue injury *in vivo*.

Periodicity of trailing edge F-actin enrichment was a prominent feature of wound-directed migration of myeloid cells, as unveiled by Fourier transformation analysis. Also of note, all PAR mutants not only impaired directional movement but also disrupted the periodicity of F-actin enrichment in the trailing edge of migrating leukocytes. Mathematical models predict that oscillatory behaviors arising from the onset of negative-feedback mechanisms in cells responding to steady stimuli show a gradual dampening of the amplitude of the response, whereas a constant amplitude might reflect the existence of a process controlled by a fast oscillatory input, which could have evolved to generate biological responses only if the input has reached a certain threshold (Cheong and Levchenko, 2010). We speculate that trailing edge F-actin oscillations in directed migration are the outcome of signaling inputs reaching critical concentration thresholds in spatially defined domains of the cells, namely the rear edge of polarized directionally migrating leukocytes.

Another hallmark of polarity in most migrating cells is the orientation of the MTOC–nucleus axis relative to the front–rear cellular axis (Luxton and Gundersen, 2011). Most *in vitro* studies, generally performed in 2D systems, showed that leukocytes position the MTOC in the uropod and behind the nucleus (Krummel and Macara, 2006; Sánchez-Madrid and del Pozo, 1999). Surprisingly, we found that the MTOC is highly dynamic in leukocytes migrating to wounds, shifting continuously from front to back orientation with respect to the nucleus. We also revealed that MTOC perinuclear mobility is PAR-complex-regulated and correlates with directional movement. As the MTOC–nucleus orientation axis is linked to the dynamics of vesicular trafficking (Luxton and Gundersen, 2011), we reason that MTOC position around the nucleus might regulate directional migration by constantly adapting microtubule nucleation and vesicle trafficking to the specific needs of 3D ameboid motility. We also found that ROCK-dependent actomyosin contraction controls MTOC perinuclear positioning, reminiscent of what happens during glial-guided neuronal migration (Solecki et al., 2009). One likely scenario would place ROCK as an upstream regulator of the PAR complex, as previously suggested for crawling cells (Nakayama et al., 2008). However, we do not favor this hypothesis because; (1) inhibiting ROCK activity does not perturb the polarized distribution of GFP–PKC-ζ during migration and (2) interfering with the catalytic function of the PAR complex reduces RhoA activity, as determined by FRET ratiometric imaging. This is consistent with studies in dendritic spine morphogenesis, which implicate the PAR complex in the control of regulators of Rho–GTP levels (Zhang and Macara, 2008).

What drives GFP–PKC-ζ polarization to the front of migrating leukocytes? Functional antagonism between PAR and Scribble complexes has been suggested in several settings to explain the spatial segregation of each of the polarity modules to opposite sides of the cells (Humbert et al., 2006). Such antagonism requires aPKC catalytic function. Importantly, in polarized T-cells *in vitro*, the PAR complex is distributed at the front of the cell, whereas the Scribble complex is localized at the tail (Krummel and Macara, 2006; Ludford-Menting et al., 2005). Further studies addressing these issues will have to involve quantitative imaging in genetically tractable living organisms, including the assessment of the stoichiometry and effector function of the molecular complexes controlling cell polarization.

## MATERIALS AND METHODS

### Fish stocks

Medaka (*Oryzias latipes*) and zebrafish (*Danio rerio*) stocks were maintained as described previously (Koster et al., 1997; Westerfield, 2000). Lines used in the study were medaka wild-type *Cab*, TG(FmpoP::memYFP) (Grabher et al., 2007) (both animals kindly provided by J.W.), TG(FmpoP::EB3-EGFP/FmpoP::RFP-Lifeact), TG(FmpoP::mCherry) and zebrafish MPO::GFP (Renshaw et al., 2006). Experimental animals were kept and treated according to the German (Tierschutzgesatz) or Italian (decree 116/92) national guidelines and experimental procedures were approved by Institutional Animal Care and Use Committee.

### Sudan Black staining and imaging

Sudan Black staining was performed in larvae as reported previously (Le Guyader et al., 2008). Images were taken with an IX81 Olympus inverted microscope equipped with an Olympus LUCPlanFLN NA 0.6/40× dry objective, coupled to a Nikon DS-5Mc-U1 digital camera and driven by Nikon NIS Elements software.

### DNA Expression Vectors

All the DNA expression vectors contained the fugu myeloperoxidase promoter fragment (FmpoP), I-*Sce*I meganuclease recognition sites and an SV40 polyadenylation sequence. The construct FmpoP::memYFP was a kind gift from J.W. (Grabher et al., 2007). Constructs with each of the following sequences in the I-*Sce*I-FmpoP backbone vector were engineered: nuclear H2AmCherry (a generous gift from J.W.) (Brown et al., 2010), Lifeact–Ruby [RFP–Lifeact; a generous gift from Michael Sixt; Institute of Science and Technology Austria (IST Austria), Klosterneuburg, Austria] (Riedl et al., 2008); EB3–EGFP (a generous gift from Virginie Lecaudey; BIOSS Center for Biological Signalling Studies, University of Freiburg, Freiburg, Germany) (Carvalho et al., 2009); nuclear H2B–CFP (a generous gift from Reinhard W. Köster; Zoological Institute, TU Braunschweig, Braunschweig, Germany) (Distel et al., 2010); monomeric Cherry (mCherry; Clontech Laboratories); cytosolic RhoA-FRET biosensor (a generous gift from Erez Raz; Institute of Cell Biology, Center for Molecular Biology of Inflammation, Münster University, Münster, Germany) (Kardash et al., 2010); FLAG-tagged rat PKC-ζ-WT or PKC-ζ-KW (generous gifts from Alex Toker; Beth Israel Deaconess Medical Center, Harvard Medical School, Boston, MA) (Chou et al., 1998) and GFP-tagged PKC-ζ-WT (GFP–PKC-ζ); mCherry–P2A-memYFP; mCherry–P2A-PKC-ζ-WT; mCherry–P2A-PKC-ζ-KW; Myc-tagged aPKC-binding region of medaka PAR-3 (PAR-3-aPKCBR) and Myc-tagged N-terminal domain of medaka PAR-6 (PAR-6-NT).

### RT-PCR and PCR

The aPKCBR of rat PAR-3 (NCBI Reference Sequence; NP_112514.1) and the N-terminal region of human PAR-6A (NCBI Accession; Q9NPB6) were as described previously (Izumi et al., 1998; Nishimura et al., 2005). The Myc-tagged corresponding regions of medaka PAR-3 (Ensemble, ENSORLP00000002648; amino acids 653–879) or medaka PAR-6B (Ensemble, ENSORLP00000002315; amino acids 1–124) were isolated by RT-PCR (Invitrogen) from wild-type cDNA. The Myc-tagged cDNAs were amplified by two rounds of PCR, using sequential sets of primers: PAR-3-aPKCBR 1st forward, 5′-GAGCGCAGAATCTCCCACTC-3′; PAR-3-aPKCBR 1st reverse, 5′-TCATCGTCGTCTTCCACAGT-3′; PAR-3-aPKCBR 2nd forward, 5′- TTTTGAATTCACCACCATGGCATCAATGCAGAAGCTGATCTCAGAGGAGGACCTGGGAGAGCGCAGAATCTCCCACTCTTTG-3′; PAR-3-aPKCBR 2nd reverse, 5′-ATAGTTTAGCGGCCGCATTCTTATCTAGGCGGGCCGGTCATAGGATTTGTC-3′; PAR-6-NT 1st forward, 5′-TTTTCTCGAGGGAATGAACAAAAACCACCGAGTGCCG-3′; PAR-6-NT 1st reverse, 5′-AAAAGAATTCCTAGGCGTCCGGCCTCAGAAGAAC-3′; PAR-6-NT 2nd forward, 5′-TTTTGAATTCACCACCATGGCATCAATGCAGAAGCTGATCTCAGAGGAGGACCTGGGAAACAAAAACCACCGAGTG-3′; PAR-6-NT 2nd reverse, 5′-ATAGTTTAGCGGCCGCATTCTTATCTAGGCGTCCGGCCTCAGAAGAAC-3′.

The GFP–PKC-ζ fusion was generated by cloning eGFP in frame at the N-terminal end of rat PKC-ζ cDNA. P2A-linked multicistronic cassettes were designed and generated based on previous reports (Szymczak-Workman et al., 2012a; Szymczak-Workman et al., 2012b). cDNAs were cloned into I-*Sce*I-FmpoP backbone vector. Sequences were confirmed by DNA sequencing.

### DNA injection

For injection of one or two constructs, a solution containing 15–22.5 ng/µl of the single plasmid DNA or 7.5–11.25 ng/µl of each construct, 1× I-*Sce*I buffer (New England Biolabs) and 0.25 units/µl I-*Sce*I meganuclease (New England Biolabs) was injected into one-cell-stage embryos (Rembold et al., 2006; Thermes et al., 2002). DNA plasmids were expressed transiently as stated in Results.

### TG(FmpoP::EB3-EGFP/FmpoP::RFP-Lifeact) and TG(FmpoP::mCherry) lines

Transgenic lines were created with I-*Sce*I-FmpoP backbone vectors as reported previously (Rembold et al., 2006; Thermes et al., 2002). Double injection of EB3–EGFP and RFP–Lifeact or single injection of mCherry was performed into one-cell-stage *Cab* embryos. Stable transgenic lines with double expression of EGFP and RFP or single expression of mCherry in myeloid cells were designated TG(FmpoP::EB3-EGFP/FmpoP::RFP-Lifeact) and TG(FmpoP::mCherry), respectively.

### Tail-fin wounding and live imaging

Imaging was performed on fish at 9–11 days post-fertilization that were wounded and mounted as reported previously (Grabher et al., 2007). Time-lapse fluorescence images were acquired with an UltraView VoX spinning-disk confocal unit (Perkin Elmer) equipped with an inverted Nikon Eclipse Ti microscope, coupled to a C9100-50 emCCD camera (Hamamatsu) and a Yokogawa CSU-X1 scanning head and driven by Volocity software (Improvision, Perkin-Elmer). CFP/YFP or GFP/RFP or Cherry imaging was performed using 405-nm, 488-nm and 568-nm laser lines and a specific multiband pass emission filter. Image sequences were generated as follows: YFP/Cherry or RFP every 20–25 s using a NA 0.75/20× objective and 1–3 µm step size; GFP/Cherry every 15 s using a NA 1.3/40× oil-immersion objective lens and 1 µm step size; CFP/GFP/RFP every 15–18 or 7–8 s using, respectively, a NA 1.3/40× or NA 1.4/60× oil-immersion objective lens and 1 µm step size. Differential interference contrast (DIC) images were taken with low-level illumination with a halogen lamp. Where indicated, embryos were pretreated with 500 µM Y-27632 (Sigma) for 2 h, and then images were taken with the drug in ERM-tricaine solution (Sigma; 0.16 mg/ml). Images were processed with ImageJ software (NIH). Upon background subtraction for each fluorescence channel, a Gaussian blur filter was applied. Brightness and contrast were set and then multi-channel image sequences were overlaid. To make overlay images of DIC and fluorescence pictures, *Z*-stacks were overlaid onto a single DIC plane.

### Coexpression analysis of two FmpoP-driven transgenes

The degree of coexpression of two transgenes was determined as the fraction of memYFP^+^ H2AmCherry^+^ cells in the fluorescent myeloid cell population.

### Cell dynamics analysis

Transgenic cells (H2AmCherry^+^) or internal control cells (H2AmCherry^−^) that were positioned at the same distance from the wound were tracked with the Manual Tracking plugin (ImageJ). The resulting 2D coordinates were analyzed with a custom-made algorithm based on MatLab Interface. Upon applying a rotation angle that centered control tracks on the *y-* (wound-directed) axis, path straightness, the *y*-directed (or wound-directed migration) speed (*Vy*) and the *x*-directed (or dispersive) speed (*Vx*) were extracted for each track and finally averaged. Straightness of the path (*S*) was defined as the ratio between the length of the segment connecting the first (t_1_) and the last (t_n_) point of the path, and the entire displacement of the cell along the *xy* plane. It was calculated as: 
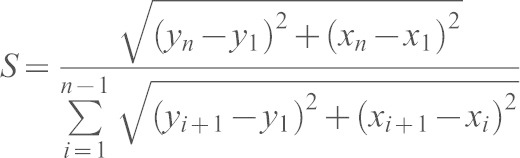


The wound-directed migration speed (*Vy*) and the dispersive speed (*Vx*) were defined as the ratio between the displacement of the cell along the *y-*axis or its absolute displacement along the *x*-axis, respectively, and the time spent by the cell during the whole migration. Values were calculated as:
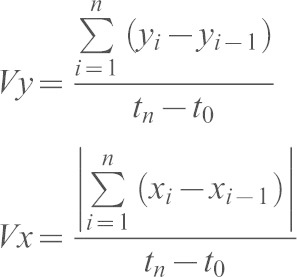


The ratio between wound-directed migration speed and dispersive speed (*Vy*/*Vx*) was determined and designated as the ‘directional speed ratio’. Speed was calculated using the Chemotaxis Tool plugin (Ibidi, Germany). Path straightness, directional speed ratio and speed were finally represented as the fold increase over the internal control values. Transgenic cells (mCherry–P2A^+^ PKC-ζ-WT^+^; mCherry–P2A^+^ PKC-ζ-KW^+^; memYFP^+^; H2B–CFP^+^ EB3–EGFP^+^ RFP–Lifeact^+^) were tracked, and the resulting coordinates were analyzed as described above, upon applying a rotation angle that centered the tailfin rays on the wound-directed axis.

### Cytoskeletal and cell shape analysis

Cell dynamics parameters were determined in an unbiased manner for all cells in the wound area. To assess shape and cytoskeletal dynamics in a more qualitative manner, control cells or a representative cohort of mutant cells displaying uncoordinated movement towards the wound site were analyzed during migration. In the analyses, the direction-of-migration vector at t_i_ corresponded to the segment connecting the centroids of the cell at t_i−1_ and t_i+1_. Measurements were carried out using ImageJ software.

### F-actin analysis

Cell shapes were subdivided in three equally long regions, ‘front’, ‘center’ and ‘back’, based upon the direction-of-migration vector (Solecki et al., 2009). Mean RFP–Lifeact fluorescence intensity values were extracted and represented as a percentage of total RFP–Lifeact fluorescence.

#### Fourier analysis

Fourier analysis was applied to RFP–Lifeact fluorescence time profiles for individual cells using the Fast Fourier Transform (FFT) algorithm (MatLab Interface). The time interval analyzed was that between the first speed-up and the last speed-down processes. In rare cases, when the two limits were not obvious from the speed–time profile, all of the temporal series was used for the analysis. For each Fourier profile, the amplitude values from the five highest peaks were normalized to the highest amplitude value among the five. Normalized values were averaged in the correspondent ranges of frequencies to create a histogram showing the relative contribution of a certain range of frequencies to RFP–Lifeact oscillations. For each cell, the strength of F-actin main frequency in the ‘back’ was defined as the amplitude value of the main Fourier peak normalized to the sum of the amplitude values from the five highest peaks.

#### F-actin polarity and total speed

The percentage of total RFP–Lifeact fluorescence was obtained as described above and normalized to memYFP fluorescence levels. Values at t_i_ were finally averaged in ranges of cellular speed (*v_i_*). Cellular speed (*v_i_*) at time t_i_ was approximated by considering the positions at t_i−1_ and t_i+1_:
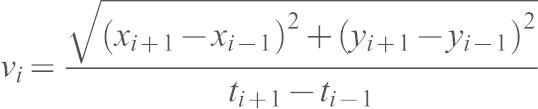


### Analysis of the MTOC and nucleus

#### Perinuclear MTOC dynamics

A vector linking the nuclear centroid and the MTOC (MTOC–nucleus vector) was determined. The angle α between the MTOC–nucleus vector and the direction-of-migration vector was defined as the MTOC/nucleus angle. Absolute values for MTOC/nucleus angles were then averaged in ranges of cellular speed (*v_i_*). The MTOC perinuclear mobility per cell was represented by the standard deviation associated with the MTOC/nucleus angles (Ranging from −180° counterclockwise to 180° clockwise).

#### MTOC and nuclear position

Analysis of MTOC and nucleus position was performed as described previously (Gomes et al., 2005).

#### MTOC and nuclear orientation

Vectors linking the centroid of the cell and the MTOC (MTOC vector) or the nuclear centroid (nucleus vector) were determined. The angle between the MTOC vector or the nucleus vector and the direction-of-migration vector was defined the MTOC angle or the nucleus angle, respectively. The MTOC and the nucleus angular mobility per cell were calculated as the MTOC perinuclear mobility.

### Analysis of F-actin distribution and MTOC or nuclear dynamics

The distance between the two extremities of the cell that cross the direction-of-migration vector was calculated (cell length). The orthogonal projection of the nuclear centroid onto the cell length was determined, and its distance from the front extremity of the cell was normalized to the cell length to give the nucleus anteroposterior position. Nucleus anteroposterior position and MTOC perinuclear orientation were then averaged in ranges of ‘back’ F-actin fluorescence.

### Morphological analysis

Roundness values during migration were obtained from cell shapes using the plugin Measurements/Shape descriptors of ImageJ.

### Ratiometric analysis of GFP–PKC-ζ

2D ratiometric images were made after *Z*-series stacking (Yoo et al., 2010). To quantify the distribution of GFP–PKC-ζ signal, we used a method based on a methodology that has been described previously (Melichar et al., 2011). We used the mCherry mask to identify the centroid of the cell and the fluorescent-weighted center of GFP–PKC-ζ∶mCherry ratiometric signal. The vector between the cell centroid and the fluorescent-weighted center was defined the asymmetry vector and its angle *α* with the direction-of-migration vector was defined as the asymmetry-migration angle.

### Imaging and analysis of RhoA activity

FRET ratiometric imaging was performed on a Leica TCS SP5 laser confocal scanner mounted on a Leica DMI 6000B inverted microscope equipped with HCX PL APO 40×/1.25–0.75 NA oil-immersion objective and driven by Leica LAS AF software. FRET sensor/mCherry were excited with violet (405-nm laser diode) and yellow (561-nm laser diode) laser lines, respectively. *Z*-stacks were collected every 15 s with 1.3 µm step size. ImageJ software was used to generate YFP∶CFP ratio images as described previously (Kardash et al., 2011). Mean emission ratio of YFP∶CFP for the entire cell during migration was calculated to determine the average activation level of RhoA.

### Statistical analyses

Statistical analyses were performed as described in the figure legends (Graphpad Prism, San Diego, CA). For circular statistics, inter-sample statistics and data visualization were performed using the Oriana software (Kovach Computing Services, Anglesey, Wales). When needed, Levene's test of homogeneity of variances was used to test for equality of variances.

## Supplementary Material

Supplementary Material
