# Supplementary material for: The PAR complex controls the spatiotemporal dynamics of F-actin and the MTOC in directionally migrating leukocytes
Source: J Cell Sci. 2014 Oct 15;127(20):4381–95. doi: 10.1242/jcs.146217 (PMC4197085; doi:10.1242/jcs.146217)
Supplement: Supplementary Material [file supp_127_20_4381__index.html]

The PAR complex controls the spatiotemporal dynamics of F-actin and the MTOC in directionally migrating leukocytes — Supplementary Material 

# The PAR complex controls the spatiotemporal dynamics of F-actin and the MTOC in directionally migrating leukocytes

## JCS146217 Supplementary Material

**Files in this Data Supplement:**

- **Supplementary Material**
