# Supplementary material for: The PAR complex controls the spatiotemporal dynamics of F-actin and the MTOC in directionally migrating leukocytes
Source: J Cell Sci. 2014 Oct 15;127(20):4381–95. doi: 10.1242/jcs.146217 (PMC4197085; doi:10.1242/jcs.146217)
Supplement: Supplementary Material [file supp_127.20.4381_JCS146217.pdf]

SUPPLEMENTARY FIGURES

SUPPLEMENTARY FIGURE LEGENDS

Fig. S1. Transient expression of transgenes in myeloid cells *in vivo*, related to Fig. 1. (A) Sudan black (SB) staining of zebrafish MPO::GFP (left panel) or medaka FmpoP::memYFP larvae (right panel), respectively at 3 or 11dpf. White arrows indicate GFP^+ SB^+ (left panel) or $memYFP^+$ SB^+ (right panel) leukocytes. Scale bars = 20 μm. (**B**) Outline of the experimental strategy used to co-express two transgenes in medaka myeloid cells. *Wild-type Cab* embryos were injected at the one-cell stage with a 1:1 mixture of DNA coding for H2AmCherry as a nuclear reporter and membrane-tethered YFP, driven by the myeloid cell-specific *Fmpo* promoter and flanked by I-SceI integration sites (Sc), in the presence of I-SceI meganuclease. **(C)** Top, illustration of the location in the fish larval tailfin imaged for the analysis of coexpression of two transgenes in live myeloid cells. Bottom, representative images of larvae containing single (YFP^+; $Cherry^+$) and double (YFP^+ $Cherry^+$) positive cells within the region highlighted. Scale bar = 50 μm. **(D)** Quantification of the degree of coexpression of two transgenes (YFP^+ $Cherry^+$ - gray bar) in live myeloid cells. Data are expressed as means ± s.e.m. of three independent experiments (173 leukocytes from 3 separate larvae). (**E**) Top, schematics of the reporter construct used to express mCherry and memYFP linked by a self-cleavable viral P2A peptide in medaka myeloid cells. Bottom, independent expression of mCherry and memYFP protein products in medaka leukocytes from the mCherry-P2A-memYFP transgene. Scale bar = 20 μm. (**F**) Schematics of the constructs used to express mCherry and PKC-ζ variants linked by a self-cleavable viral P2A peptide in medaka myeloid cells. **(G and H)** Quantitation of 2D **(G)** path straightness and **(H)** directional speed ratio (Vy/Vx) of mCherry-$P2A^+$ PKC-ζ^+ myeloid cells during the wound response. Data are expressed as means ± s.e.m. of at least two separate experiments (PKC-ζ-WT: n = 30 cells in 2 larvae, PKC-ζ-KW: n = 53 cells in 4 larvae; * $p < 0.05$, two-tailed unpaired Student's t-test).

Fig. S2. F-actin anteroposterior polarity in live myeloid cells correlates with cell speed, related to Fig. 2. **(A)** Top panel, one cycle of front-rear F-actin waves is visualized in wounded TG(FmpoP::memYFP) larvae co-expressing transiently RFP-Lifeact and PKC-ζ-WT in myeloid cells. Shown are frames taken at the indicated times from representative movies of migrating

leukocytes in the wounded tailfin. The white arrows indicate direction of migration. Scale bar = 10 μm. Bottom panel, time profile of cell's speed. Black dots correspond to the indicated time points. **(B)** Fluorescence signal distribution of F-actin in "back" and "front" regions of the cell at each time point t_i is plotted against cellular speed (determined using the positions at t_{i-1} and t_{i+1} as described in the Materials and Methods). For each range of speed, data represents means ± s.e.m. (521 counts, 16 leukocytes from 3 larvae; Back: Spearman r = 0.61, Front: Spearman r = -0.57, **** p (two-tailed) < 0.0001). **(C)** Fluorescence signal distribution in **(B)** is normalized to memYFP intensity levels and plotted against cellular speed. For each range of speed, data represents means ± s.e.m. (Back: Spearman r = 0.69, Front: Spearman r = -0.67, **** p (two-tailed) < 0.0001).

Fig. S3. MTOC and nucleus dynamics in myeloid cells migrating to wounds *in vivo*, related to Figs. 3,6. (A) Average positions of the nucleus (blue) and MTOC (green) relative to the cell centroid (defined as "0") in migrating myeloid cells. Position was normalized to cell radius calculated based on the Feret diameter (left) or based on the length of the major axis from the best fitting ellipse (right). Data represents means ± s.e.m. of all events analyzed (296 counts, 9 leukocytes from 5 larvae; **** p < 0.0001 two-tailed paired Student's t test). **(B)** Schematics of 2D analysis on MTOC and nucleus orientation during cell migration. The dashed red arrow indicates the direction of migration defined as in Fig. 2. The green arrow from the geometric center of the cell (black dot) to the MTOC is the MTOC vector whereas the blue arrow from the geometric center of the cell to the geometric center of the nucleus (white dot) is the nucleus vector. The angle α between the MTOC vector and the direction of migration vector is defined here as the MTOC angle (green arc) while the angle β between the nucleus vector and the direction of migration vector is defined here as the nucleus angle (blue arc). **(C)** Rose diagram mapping MTOC (left) or nucleus (right) orientation and the respective spatial frequency in migrating myeloid cells during wound-response (MTOC: 307 counts, Nucleus: 309 counts, 10 leukocytes from 5 larvae). Gray areas indicate "front" or "back" orientation as in Fig. 3. **(D)** Quantification of MTOC and nucleus angular mobility in migrating myeloid cells. Angular mobility of the MTOC or the nucleus *per cell* is calculated as in Fig. 3. Red lines represent means ± s.e.m. for all cells analyzed (10 leukocytes from 5 separate larvae; two-tailed paired Student's t test). **(E)** Schematics of 2D analysis on nucleus anteroposterior (A-P) positioning

during cell migration. The black line is the cell length and represents the orthogonal projection of the cell onto the direction of migration axis (dashed red arrow). The orthogonal projection of the nuclear centroid onto the cell length is defined here as the nucleus A-P position (yellow asterisk) and it approaches the value 1 when the nucleus is positioned at the "back" of the cell. **(F)** MTOC perinuclear orientation and A-P nuclear positioning are plotted against F-actin fluorescence signal distribution in the "back" region of the cell. For each range of "back" F-actin distribution, data represents means ± s.e.m. (A-P Nucleus vs "Back" F-actin - Counts: 383, Spearman r = -0.1595, ** p (two-tailed) = 0.0017; Angle MTOC/Nucleus vs "Back" F-actin - Counts: 271; Spearman r = 0.2165, *** p (two-tailed) = 0.0003). See also Materials and Methods.

Fig. S4. PAR complex regulates cellular morphology in leukocytes migrating to wounds *in vivo*, related to Figs. 4,5. (A) Morphology of representative single myeloid cells migrating in the fish larval tailfin towards a wound. Shown are outlines of myeloid cell morphology over time (CTR: 10.25 minutes; PKC-ζ-WT: 12.25 minutes; PKC-ζ-KW: 15.25 minutes; PAR-6-NT: 9.25 minutes; PAR-3-aPKCBR: 19.25 minutes). The black arrows indicate direction of migration. Scale bar = 20 µm. **(B)** Histograms show the distribution of roundness values in migrating cells (CTR: 295 counts, 9 leukocytes from 5 larvae; PKC-ζ-WT: 293 counts, 8 leukocytes from 5 larvae; PKC-ζ-KW: 384 counts, 8 leukocytes from 6 larvae; PAR-6-NT: 279 counts, 8 leukocytes from 5 larvae; PAR-3-aPKCBR: 400 counts, 8 leukocytes from 5 larvae). **(C)** Quantification of cellular roundness in migrating myeloid cells. Data represents means ± s.e.m. for all events analyzed in **(B)**. For statistical analysis see also supplementary material Table S2.

Fig. S1

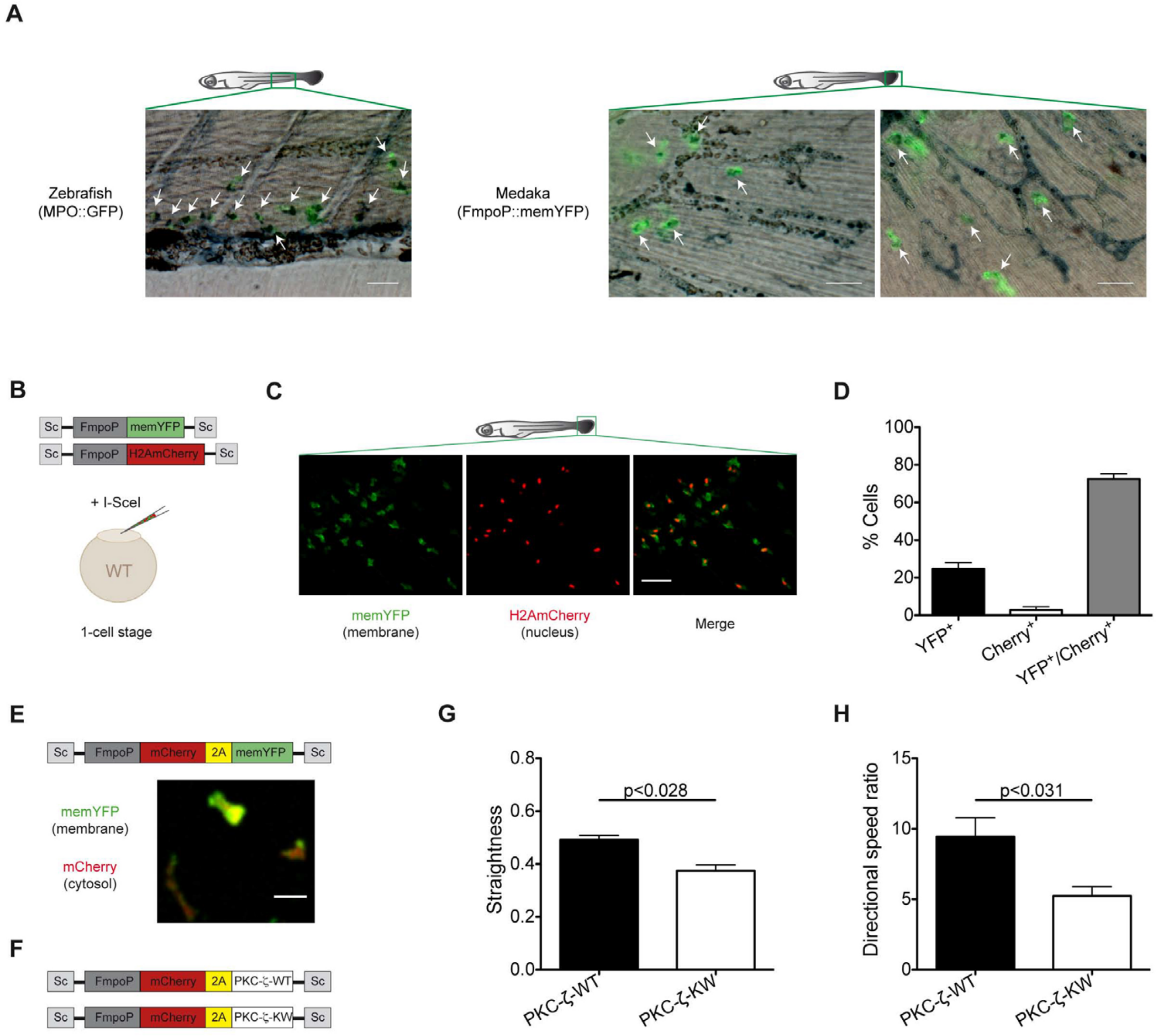

A
Zebrafish
(MPO::GFP)
Medaka
(FmpoP::memYFP)
B
Sc
FmpoP
memYFP
Sc
Sc
FmpoP
H2AmCherry
Sc
+ I-SceI
WT
1-cell stage
C
memYFP
(membrane)
H2AmCherry
(nucleus)
Merge
D
% Cells
100
80
60
40
20
0
YFP+
Cherry+
YFP+/Cherry+
E
Sc
FmpoP
mCherry
2A
memYFP
Sc
memYFP
(membrane)
mCherry
(cytosol)
F
Sc
FmpoP
mCherry
2A
PKC-ζ-WT
Sc
Sc
FmpoP
mCherry
2A
PKC-ζ-KW
Sc
G
Straightness
0.8
0.6
0.4
0.2
0.0
p<0.028
PKC-ζ-WT
PKC-ζ-KW
H
Directional speed ratio
15
10
5
0
p<0.031
PKC-ζ-WT
PKC-ζ-KW

Fig. S2

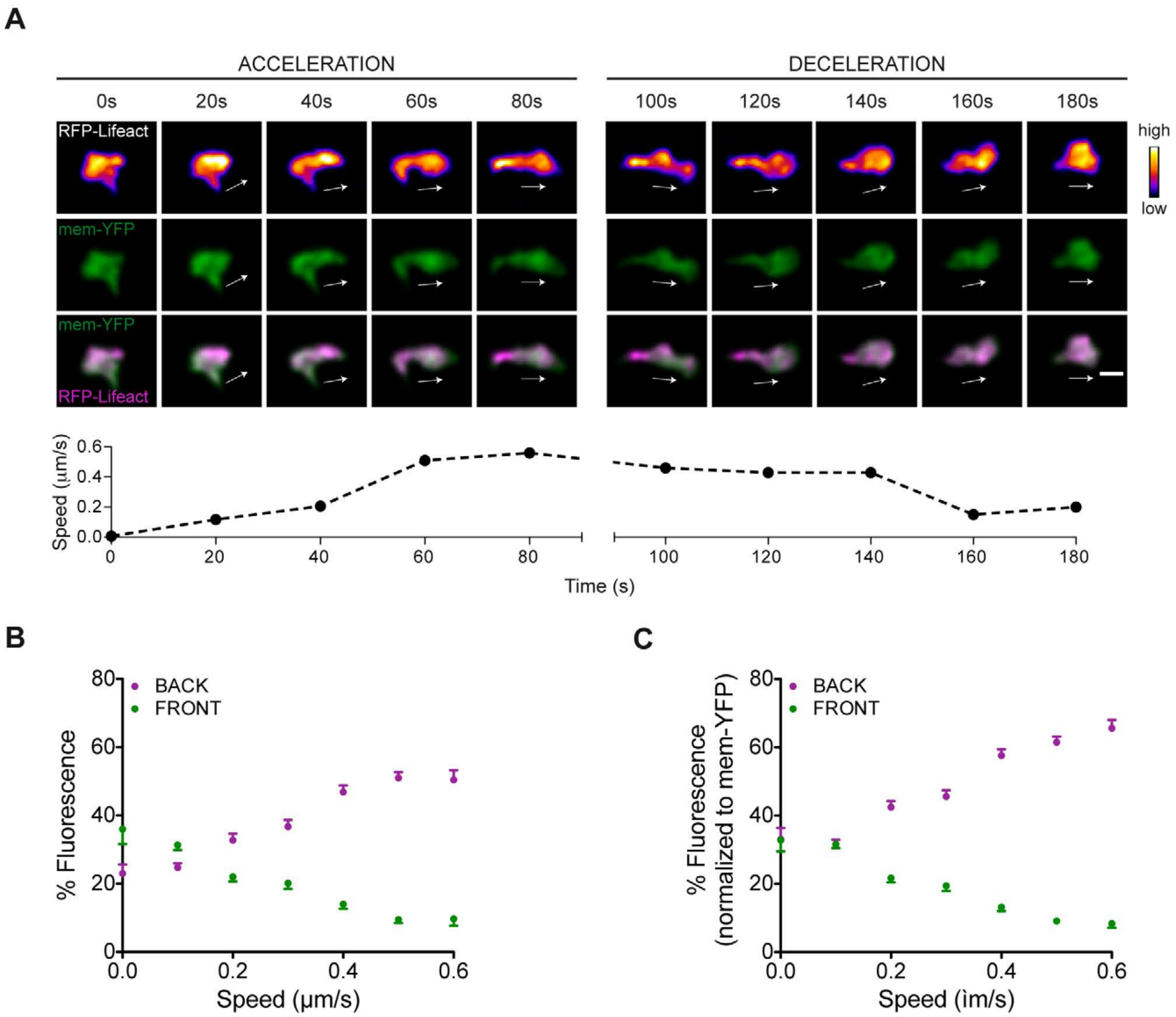
A
ACCELERATION
DECELERATION
0s
20s
40s
60s
80s
100s
120s
140s
160s
180s
RFP-Lifeact
mem-YFP
mem-YFP
RFP-Lifeact
high
low
Speed (μm/s)
Time (s)
B
BACK
FRONT
% Fluorescence
Speed (μm/s)
C
BACK
FRONT
% Fluorescence (normalized to mem-YFP)
Speed (ìm/s)

Fig. S3

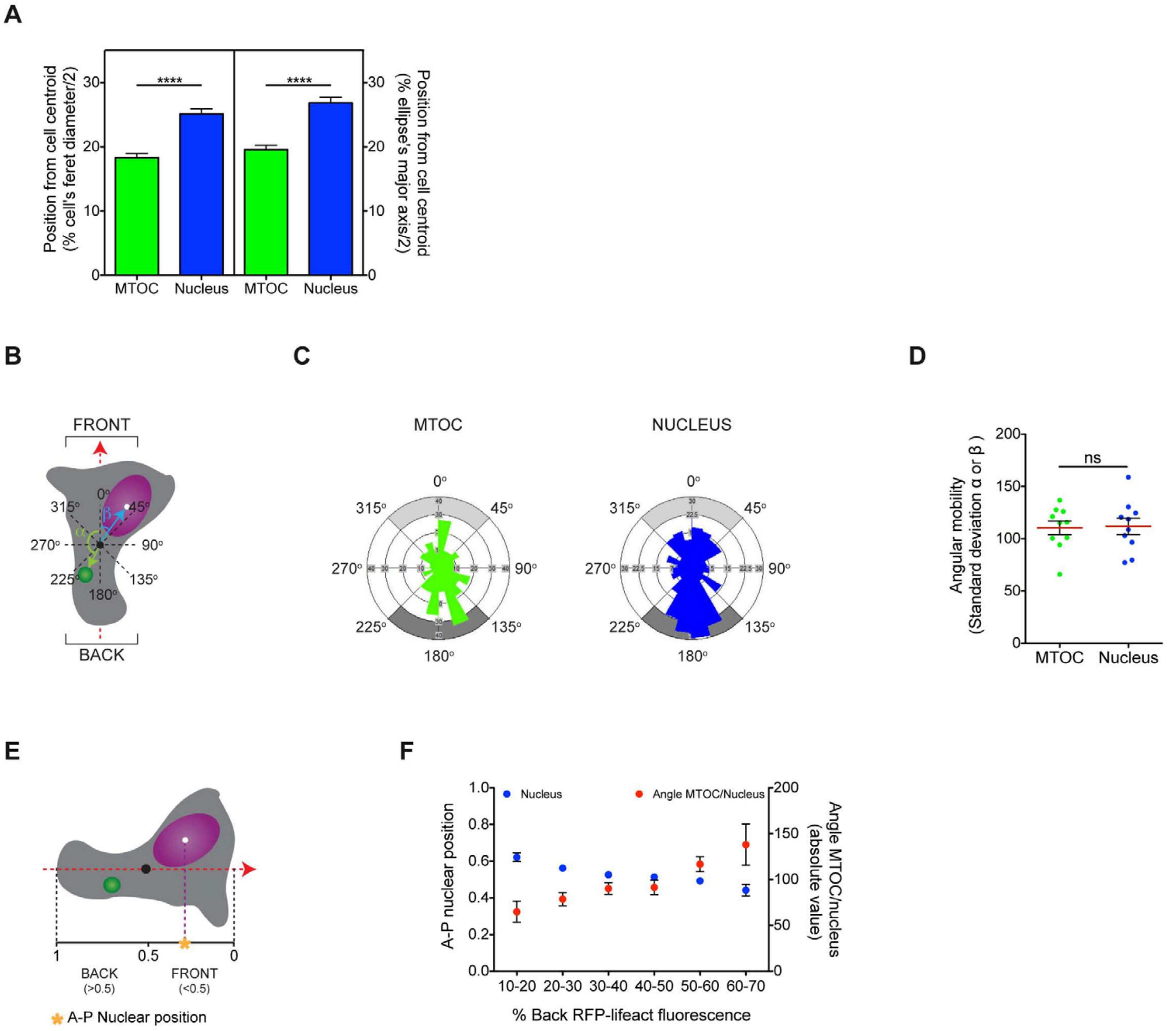
A
Position from cell centroid (% cell's feret diameter/2)
Position from cell centroid (% ellipse's major axis/2)
****
****
MTOC
Nucleus
MTOC
Nucleus
B
FRONT
BACK
0º
45º
90º
135º
180º
225º
270º
315º
α
β
C
MTOC
NUCLEUS
0º
45º
90º
135º
180º
225º
270º
315º
D
Angular mobility (Standard deviation α or β)
ns
MTOC
Nucleus
E
1
0.5
0
BACK (>0.5)
FRONT (<0.5)
A-P Nuclear position
F
Nucleus
Angle MTOC/Nucleus
A-P nuclear position
Angle MTOC/nucleus (absolute value)
10-20
20-30
30-40
40-50
50-60
60-70
% Back RFP-lifeact fluorescence

Fig. S4

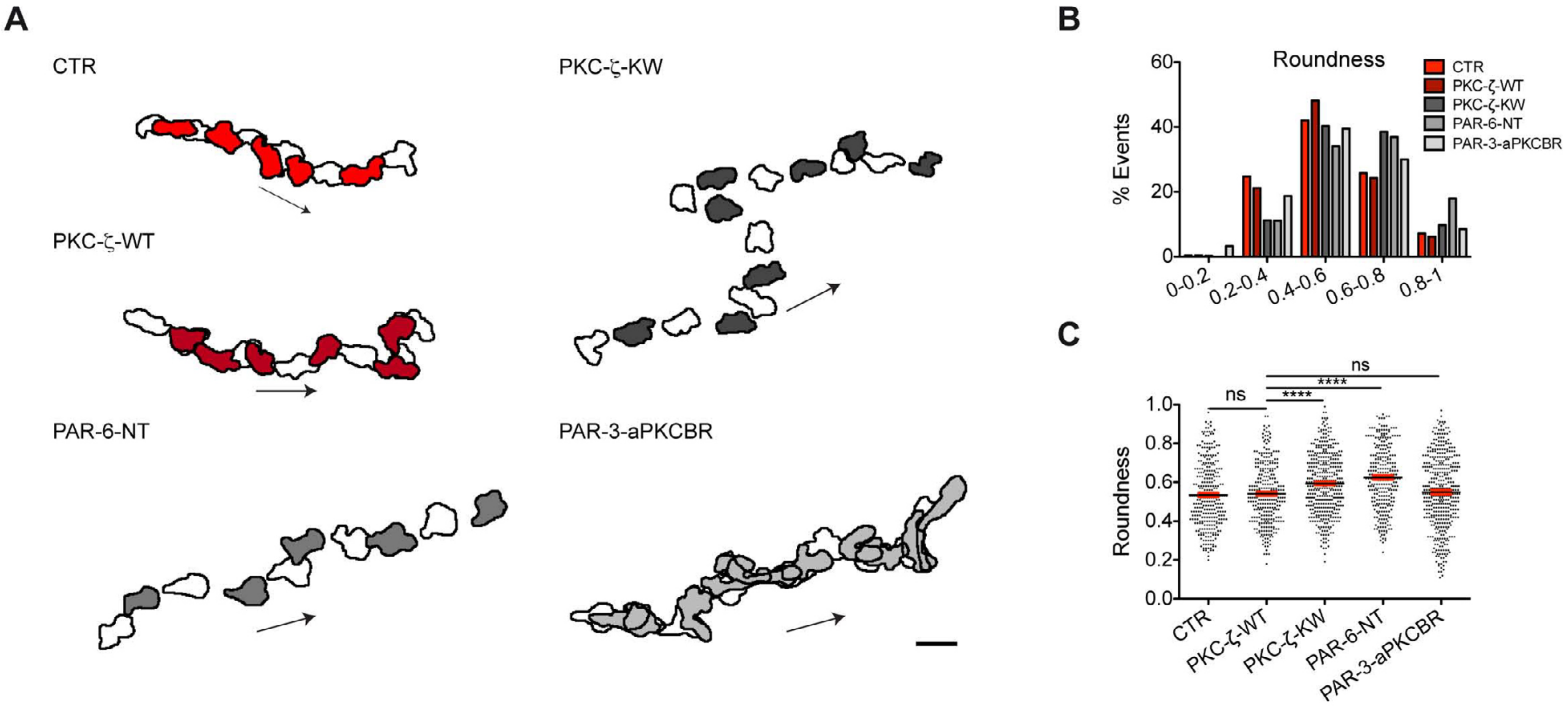
A
CTR
PKC-ζ-WT
PAR-6-NT
PKC-ζ-KW
PAR-3-aPKCBR
B
Roundness
% Events
60
40
20
0
0-0.2
0.2-0.4
0.4-0.6
0.6-0.8
0.8-1
CTR
PKC-ζ-WT
PKC-ζ-KW
PAR-6-NT
PAR-3-aPKCBR
C
Roundness
1.0
0.8
0.6
0.4
0.2
0.0
ns
****
****
ns
CTR
PKC-ζ-WT
PKC-ζ-KW
PAR-6-NT
PAR-3-aPKCBR

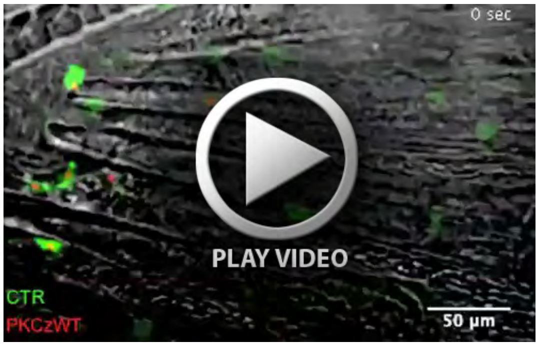

Movie 1. Effects of PKC-ζ-WT expression on myeloid cell migration to a tailfin wound. Wounding response in 11dpf TG(FmpoP::memYFP) transgenic larva co-expressing nuclear H2AmCherry and PKC-ζ-WT in memYFP$^+$ leukocytes. YFP (green), Cherry (red) images were obtained every 20 s with a spinning-disk confocal microscope. The wound is located on the bottom right hand side of the movie. Frame rate: 15 fps.

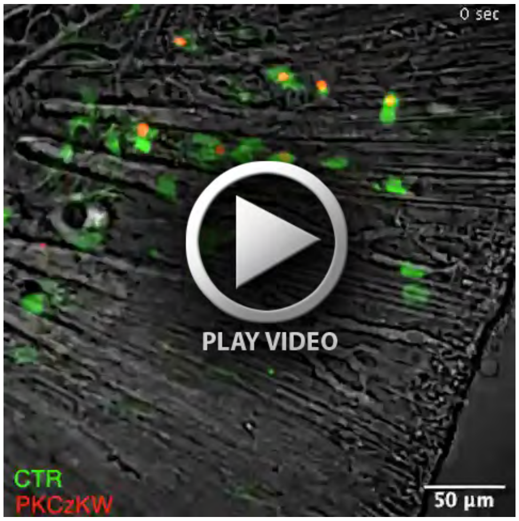

Movie 2. Effects of PKC-ζ-KW expression on myeloid cell migration to a tailfin wound. Wounding response in 11dpf TG(FmpoP::memYFP) transgenic larva co-expressing nuclear H2AmCherry and PKC-ζ-KW in memYFP$^+$ leukocytes. YFP (green), Cherry (red) images were obtained every 20.8 s with a spinning-disk confocal microscope. The wound is located on the bottom right hand side of the movie. Frame rate: 15 fps.

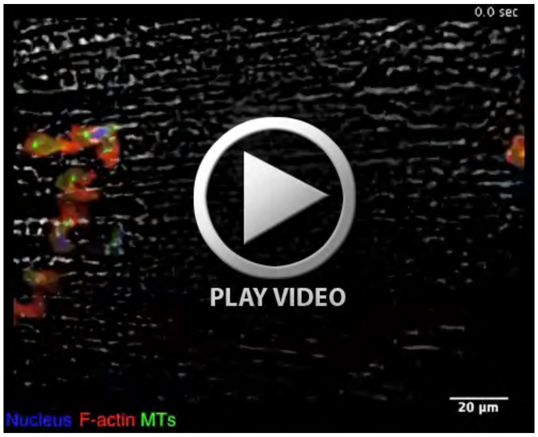

Movie 3. Imaging of cytoskeletal dynamics during wound-response. Wounding response in 9dpf TG(FmpoP::EB3-EGFP/FmpoP::RFP-Lifeact) transgenic larvae expressing nuclear H2B-CFP in myeloid cells. CFP (blue), GFP (green), RFP (red) images were obtained with a spinning-disk confocal microscope. The wound is located on the right hand side of the movie. Individual frames acquired every 16.5 s. Frame rate: 12 fps.

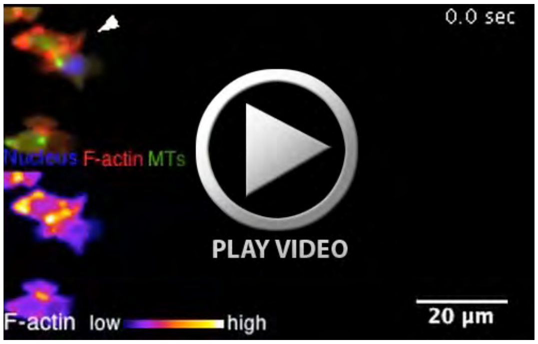

Movie 4. F-actin dynamics in leukocytes moving to wounds. F-actin dynamics of representative myeloid cells during wound-response. CFP (blue), GFP (green), RFP (red) images were obtained with a spinning-disk confocal microscope. A pseudo-color image of RFP-Lifeact was used to map intensity levels of F-actin. Blue to yellow denote low to high intensities, respectively. Wounds are located on the right hand side of the movie. **(A)** Individual frames acquired every 16.5 s. **(B)** Individual frames acquired every 8.4 s. Frame rate: 6 fps.

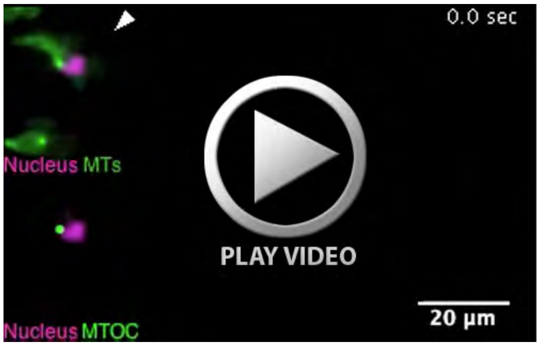

Movie 5. MTOC repositioning in leukocytes moving to wounds. MTOC positioning in the perinuclear region of cells from Movie 4. CFP (magenta), GFP (green) images were obtained with a spinning-disk confocal microscope. A digitalized reconstruction of the MTOC position (green dot) was created to illustrate MTOC orientation around the nucleus (magenta). Frame rate: 6 fps.

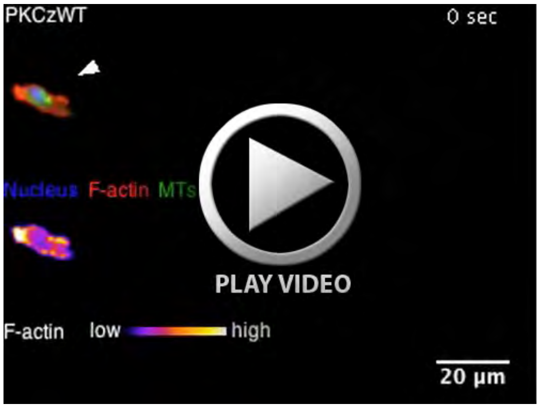

Movie 6. Effect of PAR mutants on F-actin dynamics. F-actin dynamics of representative wound-activated myeloid cells expressing PKC-ζ-WT or the different PAR-mutants. CFP (blue), GFP (green), RFP (red) images were obtained every 15-18 s with a spinning-disk confocal microscope. A pseudo-color image of RFP-Lifeact was used as in Movie 4. Wounds are located on the right hand side of the movie. Frame rate: 6 fps.

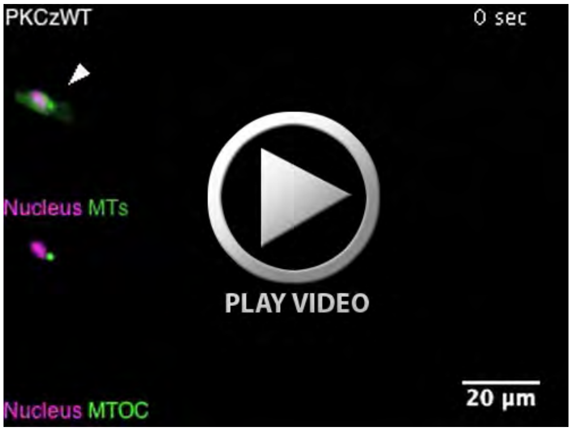

Movie 7. Effect of PAR mutants on MTOC mobility. MTOC positioning in the perinuclear region of leukocytes from Movie 6. CFP (magenta), GFP (green) images were obtained every 15-18 s with a spinning-disk confocal microscope. A digitalized reconstruction of the MTOC position (green dot) was created as in Movie 5. Frame rate: 6 fps.

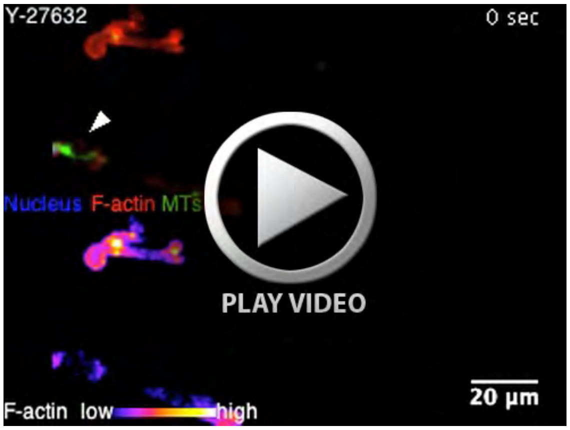

Movie 8. Perturbation of Rho signaling on F-actin dynamics. F-actin dynamics of wound-activated myeloid cells treated with Y-27632 (500 µM). CFP (blue), GFP (green), RFP (red) images were obtained every 15 s with a spinning-disk confocal microscope. A pseudo-color image of RFP-Lifeact was used as in Movie 4. Wounds are located on the right hand side of the movie. Frame rate: 6 fps.

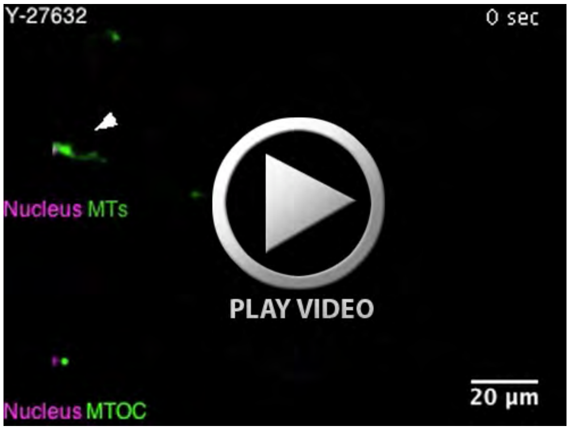

Movie 9. Perturbation of Rho signaling on MTOC dynamics. MTOC positioning in the perinuclear region of leukocytes from Movie 8. CFP (magenta), GFP (green) images were obtained every 15 s with a spinning-disk confocal microscope. A digitalized reconstruction of the MTOC position (green dot) was created as in Movie 5. Frame rate: 6 fps.

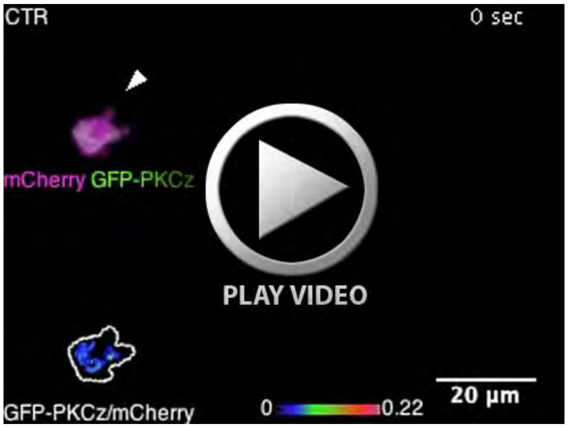

Movie 10. Effect of Rho-kinase inhibition on GFP-PKC-ζ polarity. GFP-PKC-ζ polarization in representative myeloid cells migrating to a wound in control or Y-27632-treated TG(FmpoP::mCherry) transgenic larvae, at 9dpf. GFP (green) and Cherry (magenta) images were obtained every 15 s with a spinning-disk confocal microscope. Ratio images of GFP/Cherry were generated to reveal increases in GFP-PKC-ζ fluorescence intensities. Blue to red colors denote low to high ratio values, respectively. Wounds are located on the right hand side of the movie. Frame rate: 5 fps.

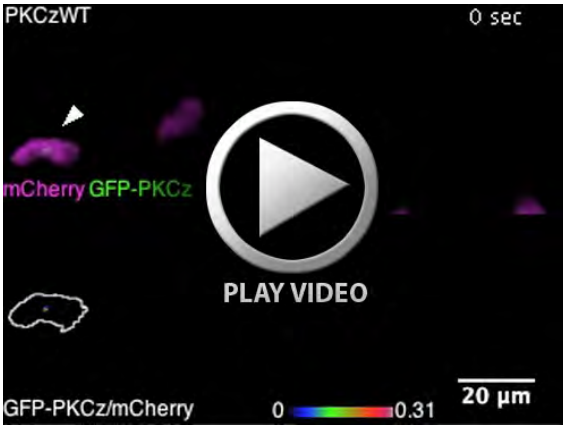

Movie 11. Perturbation of PKC-ζ activity on GFP-PKC-ζ "front" polarity. GFP-PKC-ζ polarization in representative wound-activated myeloid cells expressing PKC-ζ-WT or PKC-ζ-KW in 9dpf TG(FmpoP::mCherry) transgenic larvae. GFP (green) and Cherry (magenta) images were obtained every 15 s with a spinning-disk confocal microscope. Ratio images of GFP/Cherry were generated as in Movie 10. Wounds are located on the right hand side of the movie. Frame rate: 5 fps.

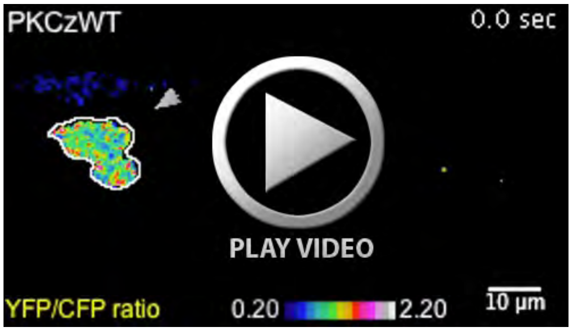

Movie 12. Effect of PKC-ζ enzymatic function on RhoA activity. RhoA activity in migrating myeloid cells co-expressing the cytosolic RhoA-FRET biosensor and PKC-ζ-WT or PKC-ζ-KW in 9dpf TG(FmpoP::mCherry) transgenic larvae. Ratio images of YFP/CFP are shown to reveal the average activation level of RhoA. Blue to white denote low to high ratio values, respectively. Wounds are located on the right hand side of the movie. Frame rate: 4 fps.

SUPPLEMENTARY TABLES

Table S1 - Summary of angle MTOC/Nucleus statistics.

Samples	Front/Back Bias		Deviation from random		Comparison (Watson test)
	Front	Back	Rayleigh test		
	%	%			
CTR	27.4	33.9	0.213	-	
PKC-ζ-WT	29.1	38.1	0.012	-	PKC-ζ-WT vs CTR: $p > 0.5$ (similar)
PKC-ζ-KW	48.1	16.4	$<10^{-12}$	+	PKC-ζ-KW vs PKC-ζ-WT: $p < 0.001$ (different)
PAR-6-NT	53.2	11.1	$<10^{-12}$	+	PAR-6-NT vs PKC-ζ-WT: $p < 0.001$ (different)
PAR-3-aPKCBR	47.7	17.4	$<10^{-12}$	+	PAR-3-aPKCBR vs PKC-ζ-WT: $p < 0.001$ (different)
Y-27632	60.9	10.7	$<10^{-12}$	+	Y-27632 versus CTR: $p < 0.001$ (different)

In the Rayleigh test for uniformity of directional data, the degree of deviation from random is given (Mardia and Jupp, 2000). Results of the Rayleigh test are displayed on a scale from random (-) to not at all random (+). In the Watson two-sample test for homogeneity of two samples of circular data, the higher the p-value, the more similar the populations (Mardia and Jupp, 2000).

Table S2 - Summary of roundness statistics.

Samples		Comparison	Means (Unpaired t test)		Homogeneity of Variances (Levene's test)	
	Mean ± SD		p value	p-value summary	p value	p-value summary
CTR	0.535 ± 0.171					
PKC-ζ-WT	0.540 ± 0.153	PKC-ζ-WT vs CTR	p=0.6950	ns (similar)	p=0.0149	* (different)
PKC-ζ-KW	0.594 ± 0.157	PKC-ζ-KW vs PKC-ζ-WT	p<0.0001	**** (different)	p=0.2400	ns (similar)
PAR-6-NT	0.624 ± 0.165	PAR-6-NT vs PKC-ζ-WT	p<0.0001	**** (different)	p=0.0732	ns (similar)
PAR-3-aPKCBR	0.549 ± 0.187	PAR-3-aPKCBR vs PKC-ζ-WT	p=0.4957	ns (similar)	p<0.0001	**** (different)

SUPPLEMENTARY REFERENCES

Mardia, K. V. and Jupp, P. E. (2000). Directional Statistics. *John Wiley and Sons, Inc.* 1–429.
